# Valorization of Agro-Industrial Lignin as a Functional Polymer for Sustainable Wastewater Treatment

**DOI:** 10.3390/polym17162263

**Published:** 2025-08-21

**Authors:** Elena Ungureanu, Bogdan-Marian Tofanica, Eugen Ulea, Ovidiu C. Ungureanu, Maria E. Fortună, Răzvan Rotaru, Irina Volf, Valentin I. Popa

**Affiliations:** 1 “Ion Ionescu de la Brad” Iasi University of Life Sciences, 3 Mihail Sadoveanu Alley, 700490 Iasi, Romania; elena.ungureanu@iuls.ro (E.U.); eugen.ulea@iuls.ro (E.U.); 2 “Vasile Goldis” Western University of Arad, 94 the Boulevard of the Revolution, 310025 Arad, Romania; ungureanu.ovidiu@uvvg.ro; 3 “Petru Poni” Institute of Macromolecular Chemistry, 41A Grigore Ghica Voda Alley, 700487 Iasi, Romania; rotaru.razvan@icmpp.ro; 4 “Gheorghe Asachi” Technical University of Iasi, 73 Prof. Dr. Docent Mangeron Boulevard, 700050 Iasi, Romania

**Keywords:** Sarkanda grass lignin, sorption, heavy metals, biopolymer, germination

## Abstract

The rational design of functional and sustainable polymers is central to addressing global environmental challenges. In this context, unmodified lignin derived from Sarkanda grass (*Tripidium bengalense*), an abundant agro-industrial lignocellulosic byproduct, was systematically investigated as a natural polymeric adsorbent for the remediation of aqueous media contaminated with heavy metals. The study evaluates lignin’s behavior toward nine metal(loid) ions: arsenic, cadmium, chromium, cobalt, copper, iron, nickel, lead, and zinc. Adsorption performance was systematically investigated under static batch conditions, optimizing key parameters, with equilibrium and kinetic data modeled using established isotherms and rate equations. Surface characterization and seed germination bioassays provided supporting evidence. Unmodified Sarkanda grass lignin demonstrated effective adsorption, exhibiting a clear preference for Cu(II) followed by other divalent cations, with lower capacities for As(III) and Cr(VI). Adsorption kinetics consistently followed a pseudo-second-order model, indicating chemisorption as the dominant mechanism. Thermodynamic studies revealed spontaneous and endothermic processes. Bioassays confirmed significant reduction in aqueous toxicity and strong metal sequestration. This work positions unmodified Sarkanda grass lignin as a bio-based, low-cost polymer platform for emerging water treatment technologies, contributing to circular bioeconomy goals and highlighting the potential of natural polymers in sustainable materials design.

## 1. Introduction

The omnipresent contamination of aquatic ecosystems by heavy metal ions, resulting largely from escalating anthropogenic activities, industrial and agricultural, represents a significant global environmental challenge [1]. These metal ions, when present at concentrations exceeding permissible limits, pose serious risks to human health, aquatic life and ecological balance [2]. Due to their inherent toxicity, non-biodegradability, and tendency to bioaccumulate within trophic chains, metallic compounds accumulate in living organisms either through direct exposure or via the food web [3]. Unlike organic pollutants, they cannot be degraded, leading to persistent and escalating ecological harm. Consequently, the development of effective, sustainable, and economically viable remediation technologies for removing these contaminants from water sources is of paramount importance [4].

Conventional methods like chemical precipitation, ion exchange, and membrane filtration, while functional, often suffer from drawbacks including high operational costs, energy demands, incomplete removal, and the generation of secondary pollutants [5]. Biosorption, utilizing naturally derived materials to bind and concentrate pollutants, has emerged as a promising alternative strategy, aligning with principles of green chemistry and environmental stewardship [6].

Plant-based materials are considered effective biosorbents due to their static binding capacity, which occurs through sorption at the cell surface, along with both intracellular and extracellular accumulation [7]. To be classified as an efficient biosorbent, the material must possess a large surface area, selectivity, sorption capacity, and compatibility with the target contaminant and the overall process [8]. Moreover, desorption capacity and biosorbent recovery are of significant importance, as it is preferable to employ biosorbents capable of undergoing multiple adsorption–desorption cycles, thereby substantially reducing the operational cost of the process [9].

In general, primary biomass and bio-derived secondary byproducts serve as effective biosorbents for the removal of heavy metals, transforming waste materials into valuable resources [7]. For instance, recent studies have successfully tested materials such as pine cones [10], cork, and pine bark [11] as biosorbents for toxic metal adsorption. Lignocellulosic biomass waste is primarily composed of cellulose, hemicellulose, and lignin [8]. In a 2023 study, Zhang and collaborators identified lignin as the key component in biomass for metal sorption, owing to its abundance of electron-donating active sites provided by its functional groups, which offer an exceptional framework for heavy metal ion binding [12].

Among the plethora of potential biomaterials, lignin stands out due to its position as the second most abundant terrestrial biopolymer after cellulose [13]. This complex, amorphous polyphenolic macromolecule, readily available as a byproduct from the pulp and paper industry and biorefineries, possesses a unique structure rich in functional groups—including hydroxyl (phenolic and aliphatic), carboxyl, carbonyl, and methoxyl fragments. These groups impart lignin with inherent potential for ion exchange and complexation, making it an attractive candidate precursor for low-cost adsorbents [14].

While various lignins from different sources and extraction methods exhibit diverse properties, a focused investigation into specific, well-characterized lignin types is important for understanding structure–performance relationships [15].

**Figure 1:**
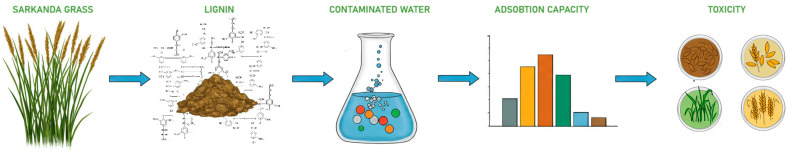
Experimental workflow for lignin-based heavy metal adsorption studies.

Sarkanda grass (*Tripidium bengalense*), an abundant lignocellulosic biomass residue, presents an opportunity for valorization within a circular bioeconomy framework [16,17]. The lignin derived from this source, utilized in its unmodified state, has been the subject of a systematic series of investigations by our group, exploring its capacity to remediate a wide spectrum of contaminants: metalloid arsenic (As) and metals such as cadmium (Cd), chromium (Cr), cobalt (Co), copper (Cu), iron (Fe), nickel (Ni), lead (Pb), and zinc (Zn) [18,19,20,21,22,23,24,25,26,27,28]. An overview of the experimental methodology is illustrated in Figure 1.

Given the environmental and public health implications associated with these elements, it is essential to consider the World Health Organization (WHO) permissible limits for heavy metals and metalloids in drinking water. These guideline values serve as critical benchmarks for assessing contamination severity and prioritizing remediation efforts. The sources of these contaminants are often anthropogenic, stemming from industrial discharges, agricultural runoff, and corrosion of infrastructure, while their toxicological profiles include a wide range of adverse effects—from nephrotoxicity and neurodegeneration to carcinogenicity and developmental disorders—underscoring the urgency of developing sustainable, lignin-based removal strategies [29].

Table 1 summarizes the WHO’s drinking-water guideline values for these contaminants alongside their principal anthropogenic and natural sources, known adverse health effects, and key literature references, providing a clear framework for evaluating lignin’s performance in sequestering regulated metal and metalloid species [29].

The contaminants chosen in the research are of critical concern due to their ubiquity in industrial, agricultural, and natural systems, coupled with their severe health risks at concentrations exceeding WHO permissible limits [29]. Common anthropogenic sources include mining effluents (Cd, Pb, Zn), electroplating (Cr, Ni), coal combustion (As), and corroded plumbing (Cu, Fe). Chronic exposure to these pollutants is linked to neurotoxicity (Pb, As), carcinogenicity (Cr^6+^, As), renal dysfunction (Cd), and systemic disorders (Cu, Ni), as outlined in the WHO Guidelines for Drinking-water Quality (2017).

Our investigations prioritize these metals due to their regulatory significance and documented persistence in aqueous environments, necessitating cost-effective remediation strategies.

**Figure 2:**
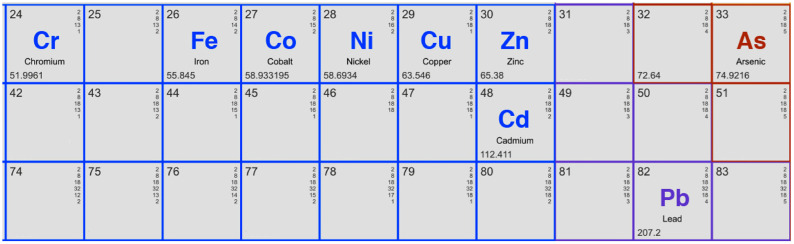
Distribution of selected contaminant elements in the periodic table.

To provide a clear visual framework for the diverse chemistry of the pollutants under investigation, Figure 2 illustrates their positions within the periodic table. This schematic is intended to offer the reader an immediate appreciation for the scope of our comparative analysis, which spans first-row transition metals (Cr, Fe, Co, Ni, Cu, Zn), heavier post-transition metals (Cd, Pb), and a metalloid (As). This visual grouping helps to contextualize the subsequent discussions on how periodic trends, such as ionic radius and electronegativity, correlate with the observed adsorption affinities on the same lignin substrate. The ability to directly compare the behavior of adjacent elements like Co, Ni, and Cu, for example, is a key strength of this study, and Figure 2 serves as a constant reference for these elemental relationships.

This review aims to synthesize and critically evaluate the findings from this coherent body of research focused exclusively on unmodified Sarkanda grass lignin as a biosorbent. We consolidate experimental data concerning its performance in removing As(III), Cd(II), Cr(VI), Co(II), Cu(II), Fe(II), Ni(II), Pb(II), and Zn(II) ions from aqueous solutions under static conditions.

Key aspects examined across these studies include the optimization of adsorption parameters (pH, adsorbent dose, contact time), detailed analysis of adsorption equilibrium using Langmuir and Freundlich isotherms, elucidation of adsorption kinetics via pseudo-first and pseudo-second-order models, interpretation of thermodynamic parameters where available, and mechanistic insights derived from these models and surface characterization.

Furthermore, the unique inclusion of biological assays using wheat and tomato seed germination provides complementary evidence for both the efficacy of pollutant removal and the sequestration of toxicity by the lignin matrix. By consolidating these results, this research article seeks to provide a comprehensive understanding of the adsorption behavior of unmodified Sarkanda grass lignin towards diverse metal(loid) species, assess its potential and limitations as a wastewater remediation agent, and identify key trends and future research directions stemming from this focused research trajectory. To achieve this, our comparative analysis is guided by two central hypotheses:

1. Hypothesis of Performance and Selectivity: We hypothesize that the unmodified Sarkanda grass lignin, owing to its distinct physicochemical properties (e.g., high density of carboxyl/phenolic groups), will exhibit significant but highly selective adsorption capacities towards a diverse suite of cationic, anionic, and neutral heavy metal(loid) pollutants.
2. Hypothesis of Mechanistic Unification: We hypothesize that despite the varied chemical nature of the pollutants, a common, underlying rate-limiting mechanism—namely chemisorption, as evidenced by pseudo-second-order kinetics—will consistently govern the adsorption process across all systems, demonstrating a unified reactive behavior of the lignin’s surface.

Considering these aspects, the present study provides a systematic comparison, based on the obtained experimental data, regarding the adsorption behaviors and mechanisms of nine metals/metalloids within a uniformly designed experimental system, utilizing consistent material sources and conditions. This approach culminates in the establishment of “adsorption selectivity rules,” as understanding the interaction between metal ions and the relationship between the metal and the biosorbent is essential for achieving effective results when applying this method under real-world conditions.

## 2. Materials and Methods

### 2.1. Materials

The foundation of the adsorption studies synthesized in this review rests upon a consistent adsorbent material: lignin derived from Sarkanda grass (*Tripidium bengalense*), an abundant herbaceous plant species. Across all investigations discussed herein [18,19,20,21,22,23,24,25,26,27,28], the unmodified Sarkanda grass lignin was sourced from Granit Recherche Development S.A., Lausanne, Switzerland (often designated Sarkanda Grass-100SA-140). The material was supplied and used in its raw, as-received form, which was a fine powder, without any further pelletization or granulation. This ensures material uniformity throughout the experimental program.

Lignin was obtained via alkali extraction as a co-product of cellulose isolation, a standard industrial delignification method. This extraction methodology is known to influence the final properties of the lignin. Key molecular weight characteristics for this material have been determined as follows: a weight-average molecular weight (Mw) of 831 g/mol, a number-average molecular weight (Mn) of 89 g/mol, and a resulting polydispersity index (PDI = Mw/Mn) of 9.34 [10,17,20,21]. The relatively low molecular weight is typical for lignins derived from annual plants like grasses, while the high PDI value of 9.34 is characteristic of technical lignins and indicates a very broad distribution of molecular sizes within the sample, ranging from small oligomeric fragments to larger polymer chains. This heterogeneity in molecular weight can, in turn, influence the material’s chemical and physical properties, such as its surface functional groups, porosity, and the accessibility of functional groups for adsorption.

#### 2.1.1. Chemical Characteristics

The efficiency of lignin as an adsorbent is intrinsically linked to its chemical structure, particularly the type and abundance of surface functional groups capable of interacting with target pollutants. Characterization data reported across the reviewed studies [16,20,21,22,28] provide key insights into the chemical nature of this specific Sarkanda grass lignin:

- Acidic Functional Groups: The material possesses a notable content of carboxyl groups (COOH), quantified at 3.3 mmol/g [19,20]. Additionally, aromatic hydroxyl (phenolic OH) groups were reported at 1.7 mmol/g [19,20]. These acidic moieties are crucial for cation adsorption, primarily through ion exchange mechanisms where protons are released upon binding of metal cations (M^n+^).
- Additional Oxygen-containing Groups: functional moieties inherent to the lignin structure include aliphatic hydroxyl groups (e.g., –CH_2_OH, –CHOH–), carbonyl groups (C=O), and methoxy groups (–OCH_3_). While aliphatic hydroxyls and carbonyls can participate in metal coordination and hydrogen bonding, methoxy groups—though less reactive—contribute to the overall polarity and hydrophilic/hydrophobic balance of the material.
- Chelating Capacity: Consistent with the presence of oxygen-containing functional groups amenable to coordination, the lignin exhibited a significant chelating capacity, reported as 67.14 meq/100 g [19,20]. This suggests a strong potential for forming stable chelate complexes with various metal ions, contributing significantly to the adsorption process, especially via mechanisms characterized by chemisorption.
- General Composition: Further compositional analysis indicated high purity, with acid-insoluble lignin content reported at 87% [20,21,22]. A low ash content of 2.2% signifies minimal inorganic contamination, while a nitrogen content of 1.2% [20,21,22] might suggest the presence of minor protein residues or inherent nitrogenous structures within the lignin framework, although specific nitrogen-containing functional groups like amines were not explicitly quantified or discussed in the context of adsorption mechanisms in these studies.

These chemical features, particularly the accessible acidic and chelating groups, prime this unmodified Sarkanda grass lignin as a promising candidate for interacting with and sequestering heavy metal ions from aqueous solutions.

#### 2.1.2. Physical Properties and Surface Morphology

Beyond the chemical functionality, the physical structure of the adsorbent plays a vital role in governing accessibility to binding sites and influencing mass transfer kinetics. Key physical properties reported for this Sarkanda grass lignin include:

- Porosity: The material exhibits a significant average porosity of 74% [19,20], suggesting an internal structure potentially accessible to adsorbate ions, which could contribute to the overall adsorption capacity beyond the external surface area.
- Particle Size: Particle size analysis indicated dimensions primarily in the micrometer range, with an average particle diameter reported as approximately 1–4 µm [19,20], although particle size distribution data suggests a broader range may exist. These dimensions have implications for diffusion pathways and settling characteristics in potential applications.

Surface Morphology: Scanning Electron Microscopy (SEM), often coupled with Energy Dispersive X-ray (EDX) analysis, contact angle measurements, was used to visualize the lignin morphology before and after metal adsorption and to confirm the presence of adsorbed metals [19,22,23,24,27,28].

**Figure 3:**
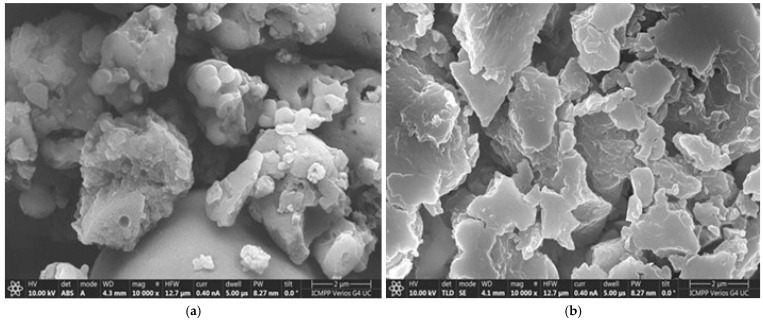
The SEM images for Sarkanda grass lignin before (**a**) and after Fe(II) adsorption (**b**), at contact time of 60 min.

Scanning Electron Microscopy (SEM) analyses, consistently presented across the studies [19,20,21,22,23], provide visual confirmation of the lignin’s morphology. Representative SEM micrographs (Figure 3) reveal particles that are somewhat irregular in shape and tend to form micrometer-sized agglomerates.

While seemingly dense in some views, the reported high porosity suggests significant internal void space within these particles or agglomerates. Optical microscopy further corroborated the particulate nature [21]. This morphology, combining an external surface with internal porosity, dictates the interface available for interaction with metal ions in solution.

From a practical application perspective, these physical characteristics are highly relevant. The particle size, ranging from 1 to 4 micrometers (µm), places this lignin powder squarely in the range of materials typically removed by microfiltration (MF) membranes, which generally have pore sizes from 0.1 to 10 µm. The reported average porosity suggests a significant internal pore network, which is advantageous for intraparticle diffusion of metal ions. However, using this fine powder directly in a flow-through system could lead to high-pressure drops or membrane fouling. Therefore, while the material’s inherent porosity and surface area are high, its direct application would be better suited for packed-bed columns (with appropriate support) or for use as a functional additive that is later removed by MF. For integration with more advanced systems like ultrafiltration (UF) membranes (pore sizes 1–100 nm), the lignin would likely need to be immobilized onto a larger support or incorporated into a composite material to prevent it from passing through or blocking the membrane pores [30].

These features present the unmodified Sarkanda grass lignin as a functional material with significant proton donating and chelating groups, high porosity, and a micro-particulate, agglomerated morphology. These inherent characteristics form the basis for its observed performance as a biosorbent for a diverse suite of heavy metal(loid) ions, as detailed in the subsequent chapters.

### 2.2. Experimental Procedure

The comparative assessment of unmodified Sarkanda grass lignin’s adsorption capabilities towards a diverse array of heavy metal(loid) ions [As(III), Cd(II), Cr(VI), Co(II), Cu(II), Fe(II), Ni(II), Pb(II), and Zn(II)] was conducted through a largely standardized experimental framework across the body of work reviewed herein [18,19,20,21,22,23,24,25,26,27,28]. This consistency in methodology is important for enabling meaningful comparisons of adsorption performance across different pollutants. This section outlines the general experimental procedures employed.

#### 2.2.1. Adsorption Methodology

All adsorption experiments were carried out under static (batch) conditions at ambient laboratory temperatures, typically 20 ± 0.5 °C or 24 ± 0.5 °C [18,19,20,21,22,23,24,25,26,27,28]. Each experiment was repeated seven times to ensure the reproducibility and reliability of the results. A consistent lignin dosage of 5 g/L (Sarkanda grass lignin) was used in most trials, based on preliminary dose-optimization studies.

In a typical procedure, a known mass of lignin was added to 20 mL of aqueous metal ion solutions with varying initial concentrations (Table 2), generally ranging from 0.1 to 1.0 mmol/L [18,19,20,21,22,23,24,25,26,27,28],. Stock solutions of each target metal ion were prepared using analytical-grade salts dissolved in distilled water: NaAsO_2_ (As(III)), CdSO_4_·8H_2_O (Cd(II)), K_2_Cr_2_O_7_ (Cr(VI)), Co(NO_3_)_2_·6H_2_O (Co(II)), CuSO_4_·5H_2_O (Cu(II)), FeSO_4_·7H_2_O (Fe(II)), NiSO_4_·6H_2_O (Ni(II)), PbSO_4_ (Pb(II)), and ZnSO_4_ (Zn(II)). Working solutions were obtained by the appropriate dilution of these stock solutions. The initial concentration ranges were selected to be representative of contaminated industrial wastewaters and to generate robust isotherm data. Specifically, for most of the divalent cations (Cd, Cu, Pb, Zn, Co, Ni, Fe), the initial concentrations studied were typically in the range of approximately 5 to 115 mg/L. For the metalloid As(III) and the anion Cr(VI), lower concentration ranges of approximately 1.5 to 15 mg/L and 5 to 52 mg/L, respectively, were investigated, reflecting their different interaction potentials and environmental concentration profiles.

The experimental conditions—particularly concentration, pH, and contact time—were selected based on a thorough review of the literature to ensure environmental relevance and to enable reliable modeling of adsorption kinetics and isotherms. Initial metal concentrations were chosen to reflect typical levels found in moderately to heavily contaminated industrial wastewaters, while remaining within the adsorption capacity range of the lignin material (Table 2).

Recognized as a critical parameter, pH was carefully optimized for each metal ion system to ensure the target species remained in the desired soluble ionic form. Experiments were generally performed under moderately to mildly acidic conditions (pH 5–6.5; Table 3), which favor interactions with the functional groups of lignin and prevent precipitation as metal hydroxides that typically occurs at higher, alkaline pH levels.

The pH range was chosen because (1) the metal ions predominantly exist as free M^2+^ species, minimizing hydrolysis and precipitation that typically occur above pH 7, and (2) key functional groups on lignin (carboxyl and phenolic hydroxyls) are sufficiently deprotonated to enable effective binding, without the interference of excess protons as in strongly acidic conditions. Similarly, a more acidic pH was used for Cr(VI), and a near-neutral pH was applied for As(III), in accordance with the optimal species distribution and adsorption behavior reported in the literature.

After adjusting pH, lignin was added to the metal solutions, and the mixtures were left to equilibrate for predetermined contact times, typically 30, 60, and 90 (or 120) minutes (Table 3).

For most of the studied metal(loid)s, adsorption equilibrium—or near-equilibrium—was typically reached within approximately 60 min of contact time, beyond which further uptake was negligible, suggesting saturation of the readily accessible and reactive binding sites on lignin. Following equilibration, solid–liquid separation was performed by filtration.

#### 2.2.2. Analytical Techniques for Metal Ion Quantification

The residual concentration of the metal ion in the filtrate was determined using appropriate analytical methods to assess the amount adsorbed by the lignin (Table 4).

In all cases, quantification was performed against calibration curves prepared with standard solutions of the respective metal ions. The amount of metal adsorbed per unit mass of lignin (q, mg/g) was then calculated using the mass balance equation:

q = (*C_i_* − *C_e_*)*V*/*m*, (mg/g) (1)

where *C_i_* is the initial metal ion concentration (mg/L), *C_e_* is the equilibrium metal ion concentration in the filtrate (mg/L), *V* is the volume of the solution (L), and *m* is the mass of the adsorbent (g).

#### 2.2.3. Equilibrium and Kinetic Modeling

To understand the adsorption equilibrium and dynamics, the experimental data were consistently analyzed using well-established models:

- Equilibrium Isotherms: The Langmuir and Freundlich isotherm models were universally applied to describe the equilibrium distribution of metal ions between the aqueous and solid phases [18,19,20,21,22,23,24,25,26,27,28]. The goodness-of-fit was typically evaluated based on the correlation coefficient (R^2^) of the linearized forms of these models.
- Adsorption Kinetics: The pseudo-first-order Lagergren model and the pseudo-second-order Ho-McKay model were employed to investigate the rate and mechanism of adsorption [18,19,20,21,22,23,24,25,26,27,28]. Again, R^2^ values were used to determine the best-fitting kinetic model.

### 2.3. Supporting Analyses

Beyond the core adsorption experiments, several supporting analyses were frequently conducted:

- Surface Morphology: Scanning Electron Microscopy (SEM), often coupled with Energy Dispersive X-ray (EDX) analysis, and contact angle measurements, were used to visualize the lignin morphology before and after metal adsorption and to confirm the presence of adsorbed metals [13,16,17,18,20,21].
- Thermodynamic Studies: For some metal ions: Cr(VI), Cd(II), and Cu(II) (Cr, Cd, Cu), thermodynamic parameters (ΔG, ΔH, ΔS) were determined to assess the spontaneity and nature of the adsorption process [20,22,24].

### 2.4. Methodology: Seed Germination and Seedling Growth Assays

Seed germination assays using *Triticum aestivum* L. (wheat, Glosa variety) and *Lypercosium esculentum* (tomato, San Marzano variety) were performed with both the metal-loaded lignin and the filtrate to provide a biological assessment of toxicity removal and metal sequestration [13,16,17,18,20,21]. The standardized methodology involved exposing wheat seeds to two distinct sample types derived from the adsorption experiments:

1. Filtrates: The aqueous phase remaining after the separation of lignin post-adsorption.
2. Contaminated Lignin: The solid lignin adsorbent after it had been loaded with the target metal ion.

Control groups typically involved distilled water (for filtrate comparison) and uncontaminated lignin (for comparison with metal-loaded lignin). Key parameters monitored over a seven-day period usually included the number of germinated seeds (often assessed at 3 days for germination energy and 7 days for germination faculty/capacity), and sometimes the length and mass of the resulting seedlings.

## 3. Results and Discussion

The comprehensive suite of studies conducted on unmodified Sarkanda grass lignin provides a rich dataset for evaluating its adsorption performance towards a range of environmentally significant heavy metal(loid) ions: As(III), Cd(II), Cr(VI), Co(II), Cu(II), Fe(II), Ni(II), Pb(II), and Zn(II). This section systematically presents the findings related to the optimization of experimental conditions, equilibrium adsorption capacities, and the kinetics of the adsorption processes.

### 3.1. Optimization of Experimental Conditions

#### 3.1.1. Influence of Adsorbent Dose

The quantity of adsorbent relative to the volume of solution dictates the total number of available binding sites. Dose optimization studies consistently revealed that an adsorbent concentration of 5 g/L of Sarkanda grass lignin was optimal or highly effective for the removal of all investigated metal ions under the tested concentration ranges [18,19,20,21,22,23,24,25,26,27,28]. While increasing the adsorbent dose generally increases the total number of available binding sites, leading to higher percentage removal, the adsorption capacity per unit mass of adsorbent (‘q’, mg/g) often decreases beyond a certain point due to site saturation, particle aggregation, or overlapping of binding sites. Thus, the 5 g/L dose appeared to strike an equilibrium between the efficient removal and the effective utilization of the adsorbent material.

#### 3.1.2. Critical Role of Solution pH and Contact Time

The pH of the aqueous solution is a critical factor influencing both the speciation of metal ions and the surface charge characteristics of the adsorbent. For the divalent cations (Cd(II), Co(II), Cu(II), Fe(II), Ni(II), Pb(II), and Zn(II)), adsorption was generally favored in the weakly acidic to near-neutral pH range, typically between pH 5.0 and pH 6.5. At lower pH values, increased proton concentration (H^+^) likely competes with metal cations for active binding sites on the lignin (e.g., protonated carboxyl and phenolic hydroxyl groups), leading to reduced uptake. As pH increases within this range, deprotonation of these acidic functional groups occurs, creating negatively charged sites (e.g., -COO^−^, -O^−^) that facilitate electrostatic attraction and ion exchange with the positively charged metal ions. Beyond pH ~6.5–7.0, the risk of metal hydroxide precipitation becomes a concern, confounding true adsorption measurements, hence this range was generally avoided. For instance, optimal pH values were reported as:

- pH 5 for Co(II) [17], Cr(VI) [21,22], Cu(II) [24,25], Ni(II) [27].
- pH 6 for As(III) [18], Pb(II) [28], Zn(II) [28].
- pH 6.2 for Cd(II) [19,20].
- pH 6.5 for Fe(II) [26].

For Cr(VI), which exists as oxyanions (e.g., HCrO_4_^−^, CrO_4_^2−^), adsorption was favored under acidic conditions (pH 5) [21,22]. This is typical, as a lower pH promotes protonation of lignin’s surface functional groups, leading to favorable electrostatic interactions with anionic chromate species. At the same time, for As(III), which exists predominantly as the neutral H_3_AsO_3_ species at pH 6, the chosen pH of 6 was based on the literature and previous findings for optimal arsenic removal and stability of the species [18].

In conclusion, pH plays a crucial role in metal biosorption by influencing both the availability of metal ions and the surface charge of the biosorbent. Understanding these interactions is key to optimizing biosorption efficiency.

The time allowed for interaction between the adsorbent and adsorbate is crucial for achieving equilibrium. The time required to reach adsorption equilibrium is a crucial parameter for practical applications. Across all nine metal(loid) systems, adsorption was observed to be relatively rapid in the initial stages, followed by a slower approach to equilibrium.

A contact time of approximately 60 min was consistently identified as sufficient to achieve, or be very close to, adsorption equilibrium under the experimental conditions employed [18,19,20,21,22,23,24,25,26,27,28]. Extending the contact time to 90 or 120 min generally resulted in only marginal or negligible increases in metal uptake, indicating that most accessible binding sites were occupied within the first hour.

### 3.2. Equilibrium Studies: Adsorption Isotherms and Capacity

Adsorption isotherms describe the equilibrium relationship between the concentration of adsorbate in the solution and the amount adsorbed on the solid phase at a constant temperature. The Langmuir and Freundlich models were universally applied.

The suitability of the Langmuir and Freundlich models varied somewhat depending on the specific metal ion:

- Freundlich Model Predominance: For many of the metal ions, including As(III) [18], Cd(II) [19,20], Co(II) [23], Fe(II) [26], and Ni(II) [27], the Freundlich isotherm generally provided a better fit to the experimental data (higher R^2^ values) compared to the Langmuir model. This suggests that the adsorption primarily occurs on a heterogeneous surface with a non-uniform distribution of binding energies, which is characteristic of complex biopolymers like lignin, and that the adsorption of these species onto the heterogeneous surface of Sarkanda grass lignin, possibly involving multilayer adsorption or sites with varying binding energies, is well described by this empirical model.
- Comparable Fits/Langmuir Applicability: For Cr(VI) [21,22], Cu(II) [24,25], Pb(II), and Zn(II) [28], both models often described the data well, with R^2^ values being high and comparable for both ((R^2^ > 0.98)). In some of these cases, the Langmuir model also showed good applicability, implying that monolayer adsorption on a finite number of energetically equivalent sites might also contribute significantly to the overall uptake.

The Langmuir model estimates the theoretical maximum monolayer adsorption capacity (q_max_). Using data obtained at a contact time of 60 min, the q_max_ values for unmodified Sarkanda grass lignin toward the various metal(loid) ions were determined and are summarized in Table 5. These results were used to evaluate the first hypothesis concerning the adsorbent’s overall performance and selectivity. Equilibrium adsorption capacities for all nine target pollutants are likewise presented in Table 3 for direct comparison.

A detailed analysis of the data presented in Table 3 provides further insight into the adsorption behavior. For the majority of the divalent cations (Ni, Co, Pb, Zn, Fe, and Cd), the highest experimentally observed adsorption capacity (q_e_) is very close to the calculated Langmuir maximum capacity (q_max_). This proximity suggests that the experimental concentration ranges used were sufficient to approach or achieve the effective saturation of the adsorbent’s monolayer, validating the q_max_ values as a realistic measure of the material’s potential for these ions.

In contrast, a significant divergence is observed for Copper (Cu II), where the highest measured q_e_ (11.44 mg/g) is only about half of the theoretical q_max_ (22.12 mg/g). This indicates that while the affinity for Cu(II) is exceptionally high, the experiments did not explore concentrations high enough to fully saturate the available binding sites. Therefore, the q_max_ value for Cu(II) represents a significant untapped potential for this adsorbent. For As(III) and Cr(VI), the observed and theoretical capacities are both low and very similar, confirming a limited number of active sites for these species on the unmodified lignin.

The data clearly show a performance trend:

Cu(II) >> [Ni(II) ≈ Co(II) ≈ Cd(II) ≈ Pb(II) ≈ Zn(II) ≈ Fe(II)] >> [As(III) ≈ Cr(VI)].

Sarkanda grass lignin exhibits the highest affinity for Cu(II) ions. The other divalent cations (Cd, Co, Fe, Ni, Pb, and Zn) demonstrate remarkably similar and significant adsorption capacities, all falling within a tight range of ~13–14 mg/g. In stark contrast, the metalloid As(III) and the anion Cr(VI) show considerably lower adsorption capacities, indicating fewer or less favorable binding interactions with this lignin under the tested conditions.

To explore deeper into the nature of these interactions and to further explain the observed selectivity, a detailed analysis of the isotherm model parameters is crucial. Table 6 summarizes the key parameters extracted from the Langmuir and Freundlich models for all nine pollutants at the optimal 60 min equilibrium time.

For systems where the Freundlich model provided a good fit, the parameters ‘K_F_’ (Freundlich capacity constant) and ‘n’ (adsorption intensity) offer further insights. ‘K_F_’ values generally correlated with the observed adsorption capacities.

The Freundlich parameters provide further insight into adsorption capacity and surface heterogeneity. The Freundlich capacity constant, K_F,_ generally aligns with the trends observed for q_max_, with the divalent cations (e.g., K_F_ for Ni(II) = 2.008, Cu(II) = 2.000) showing higher values than As(III), Cd(II), and Cr(VI) (K_F_ ~1.0–1.1).

The Langmuir constant, **K_L_**, is directly related to the affinity of the binding sites, with a higher K_L_ value indicating a stronger interaction. The data in Table 6 reveal a striking and scientifically significant trend that differentiates two distinct types of binding behavior on the lignin surface.

The **K_L_** values for As(III) (0.315 L/mg), Cd(II) (0.341 L/mg), and Cr(VI) (0.315 L/mg) are substantially higher—by a factor of four to five—than those for the other divalent cations like Cu(II) (0.064 L/mg), Pb(II) (0.076 L/mg), and Ni(II) (0.080 L/mg). This counter-intuitive result suggests a dual-site nature for the adsorbent. While the total number of binding sites for As(III), Cd(II), and Cr(VI) is very low (as confirmed by their low q_max_ values in Table 5), the few sites that are available possess a very high intrinsic affinity for these specific pollutants. Conversely, the lignin surface possesses a large population of more moderate-affinity sites that are readily available for the other divalent cations (Cu, Pb, Ni, Co, Fe, Zn), leading to their much higher overall adsorption capacities. This nuanced understanding, derived from the **K_L_** parameters, is a key finding that goes beyond a simple comparison of maximum capacities.

The ‘1/n’ heterogeneity factor values were consistently between 0 and 1 (e.g., As(III) ~0.55 [18]; Cd(II) ~0.9 [19]; Co(II) ~0.93 [23]; Ni(II) ~0.9 [27]; Fe(II) ~0.91 [26]; Pb(II)/Zn(II) ‘n’ values >1 imply ‘1/n’ < 1 [28]), indicating favorable adsorption processes. Values of ‘1/n’ closer to 1 suggest more homogeneous binding energies and adsorption affinity, while smaller values indicate greater heterogeneity. The good fit to the Freundlich isotherm for many of these cations [19,23,26,27] suggests a heterogeneous surface with a range of binding site energies, which is expected for a complex polymer like lignin where functional groups are present in diverse chemical media.

### 3.3. Kinetic Studies: Adsorption Rate and Mechanism Pathway

Understanding the mechanisms by which Sarkanda grass lignin interacts with and retains various heavy metal(loid) ions is crucial for optimizing its application and potentially for guiding future modifications. The consistent observation of pseudo-second-order kinetics across all nine pollutants studied [18,19,20,21,22,23,24,25,26,27,28] strongly suggests that chemisorption, involving valence forces through the sharing or exchange of electrons, plays a dominant role in the rate-limiting step of these adsorption processes. This chapter synthesizes the evidence from the reviewed studies to propose and discuss the likely adsorption mechanisms for different categories of metal(loid) ions.

The kinetics of adsorption describes the rate at which metal ions are removed from solutions and provides insights into the controlling mechanisms, which is important for designing efficient adsorption systems.

Across all nine metal(loid) ions studied—As(III), Cd(II), Cr(VI), Co(II), Cu(II), Fe(II), Ni(II), Pb(II), and Zn(II)—the pseudo-second-order kinetic model consistently provided a superior fit to the experimental data compared to the pseudo-first-order model [18,19,20,21,22,23,24,25,26,27,28]. This was demonstrated by model R^2^ values that were frequently equal to or very close to 1.00 in many instances.

These results strongly support our second hypothesis, which proposed a unified adsorption mechanism across all pollutants onto unmodified Sarkanda grass lignin. The consistent alignment of kinetic data with the kinetic model suggests that chemisorption is the common rate-limiting step, regardless of the chemical differences among the ions. The model assumes that the adsorption rate is proportional to the square of the number of unoccupied active sites and typically implies a mechanism involving valence forces, such as electron sharing or exchange between the lignin surface and the adsorbed species.

Such behavior suggests that the uptake process is primarily governed by the formation of chemical bonds between the metal(loid) ions and the active functional moieties inherent to the lignin macromolecule, rather than being limited by bulk diffusion or solely by weak physical attraction. This consistent finding across a broad range of pollutants with differing chemical properties (anionic, cationic, metalloid) underscores a fundamental characteristic of the interaction between Sarkanda grass lignin and these species.

While the Ho–McKay model was universally applicable, the calculated pseudo-second-order rate constants (k_2_, g/(mg·min)) exhibited some variability across the different metal(loid) ions, reflecting differences in the intrinsic speed of the chemisorption reaction. Notably, As(III) displayed an exceptionally high k_2_ value, suggesting a very rapid chemical interaction despite its relatively low overall adsorption capacity. For the divalent cations, the rate constants were all within the same order of magnitude, indicating broadly comparable intrinsic reaction speeds for their chemical binding to the lignin surface once any initial mass transfer limitations are overcome.

Regarding the adsorption equilibrium time, although not directly a parameter derived from the kinetic models themselves, the underlying experimental data consistently showed that the process was characterized by a relatively rapid initial uptake phase, followed by a slower approach to equilibrium. For most of the metal(loid)s studied, equilibrium or near-equilibrium conditions were typically achieved within approximately 60 min of contact time. Beyond this duration, further increases in adsorption were generally negligible, indicating the saturation of the readily accessible and reactive binding sites on the lignin.

#### 3.3.1. Proposed Mechanisms for Divalent Cations (Cu(II), Ni(II), Co(II), Cd(II), Pb(II), Zn(II), and Fe(II))

For the seven divalent cations investigated, which generally exhibited higher adsorption capacities (especially Cu(II)), the primary mechanisms are likely a combination of ion exchange and complexation/chelation, consistent with the chemosorptive nature indicated by the pseudo-second-order kinetic model.

- Ion Exchange: This is a prominent mechanism explaining the high uptake of divalent cations on the Sarkanda grass lignin studied by stoichiometric ion exchange. This process specifically involves the acidic carboxyl and phenolic hydroxyl groups characterized in Section 2.1. At the operational pH range of 5–6.5, these groups are sufficiently deprotonated to facilitate a direct exchange of surface protons (H^+^) for metal cations (M^2+^) from the solution, as represented by the following equilibrium reactions:

$$2\;R\text{–}\mathrm{COOH}+M^{2+}\;↔{\left(R\text{–}\mathrm{COO}\right)}_{2}M\;+\;2H^{+}$$

$$2\;R\text{–}\mathrm{ArOH}+M2^{+}\;↔{\left(R\text{–}\mathrm{ArO}\right)}_{2}M\;+\;2H^{+}$$

This specific binding reaction is a cornerstone of the chemisorption process indicated by our kinetic data.

The observed increase in adsorption with increasing pH (up to the point of metal hydroxide precipitation) supports the involvement of lignin’s acidic functional groups, as higher pH promotes their deprotonation and enhances their capacity to bind metal ions [13,17,19,20,21,22]. This adsorption process can be conceptualized as a two-step interaction. Initially, hydrated cations are attracted to the negatively charged lignin surface via long-range, non-specific electrostatic forces, resulting in the formation of outer-sphere complexes. This is followed by a slower, rate-limiting chemisorption step, in which desolvation occurs and direct coordination bonds form between the metal ions and the lignin’s functional groups, producing more stable inner-sphere complexes. The endothermic nature of adsorption (ΔH > 0) observed for Cu(II), Cd(II), and other divalent cations [14,18] is consistent with the energy required for this desolvation step. It also reflects the disruption of existing hydrogen bonds on the lignin surface and the reorganization of solvation shells, which are necessary precursors to inner-sphere complex formation.

- Complexation/Chelation: Metal ion binding to lignin is largely governed by coordinate interactions with electron-donating functional groups, particularly oxygen atoms in hydroxyl, carboxyl, and carbonyl moieties. These interactions lead to the formation of surface complexes, often involving chelation—a process where a metal ion binds simultaneously to multiple donor atoms within the same lignin macromolecule. Such binding contributes to the chemisorptive nature of the process, which cannot be fully explained by simple ion exchange alone. Adsorption likely proceeds through an initial, non-specific electrostatic attraction between hydrated metal cations and the negatively charged lignin surface, forming outer-sphere complexes. This is followed by a slower, rate-determining transition to inner-sphere complexes, in which direct, specific coordination bonds are established. Chelation plays a central role in this step, with multidentate binding sites on lignin facilitating the formation of stable five- or six-membered rings. Notable examples include coordination between a carboxyl and adjacent hydroxyl group or between neighboring phenolic hydroxyl and methoxyl groups in guaiacyl units. Lignin’s high chelating capacity (67.14 meq/100 g) underscores its affinity for such metal–ligand interactions. This is particularly evident in the case of Cu(II), which demonstrates the highest adsorption capacity (q_max_ = 22.12 mg/g) among the studied divalent cations. Its superior uptake is likely due to a combination of its favorable ionic radius, higher electronegativity, and strong preference for forming stable chelates with oxygen-containing ligands—factors consistent with the Irving–Williams series [25].

The consistent good fit to the Freundlich isotherm for many of these cations [13,17,20,21] suggests a heterogeneous surface with a range of binding site energies, which is expected for a complex polymer like lignin where functional groups are present in diverse chemical environments.

#### 3.3.2. Proposed Mechanisms for Cr(VI)

Chromium(VI) exists as oxyanions (predominantly HCrO_4_^−^ at pH 5, the optimal pH identified for its adsorption [21]). The mechanism for its removal by Sarkanda grass lignin is distinct from that of cations:

- Electrostatic Attraction: At acidic pH (e.g., pH 5), lignin’s surface functional groups (like carboxyls and even some hydroxyls or trace amines, if present) can become protonated, leading to a net positive surface charge. This facilitates electrostatic attraction with the negatively charged chromate species.
- Ligand Exchange/Complexation: Chromate ions might directly complex with protonated functional groups or displace weaker ligands (like water) from these sites.
- Reduction to Cr(III) followed by Cationic Adsorption: Lignin, being a phenolic polymer, possesses reducing capabilities. It is plausible that some Cr(VI) is reduced to the less toxic Cr(III) by the lignin. The resulting Cr(III) cations could then be adsorbed via the ion exchange or complexation mechanisms described for other cations in the previous paragraph. The paper on biological testing of Cr(VI) loaded lignin [16] mentions “*formation of free radicals during the reduction of Cr(VI) to Cr(III)*” as a possible toxic action, indirectly supporting this redox pathway.

The surface charge of lignin depends on the solution pH, as many of its functional groups—such as carboxyl and phenolic hydroxyl groups—can gain or lose protons. At lower pH values, such as pH 5 (commonly reported as optimal for Cr(VI) adsorption), these groups tend to become protonated, giving the lignin surface a more positive character. This promotes electrostatic attraction between the lignin and negatively charged species like HCrO_4_^−^, enhancing adsorption [26].

The very low q_max_ of 0.861 mg/g for Cr(VI) [21] suggests that the number of sites favorable for anionic interaction or effective reduction/complexation is limited on this unmodified lignin under the tested conditions.

#### 3.3.3. Proposed Mechanisms for Metalloid As(III)

Arsenic(III) primarily exists as the neutral arsenous acid species (H_3_AsO_3_) at the optimal adsorption pH of 6 identified for this study [19]. This neutrality means electrostatic interactions are less likely to be dominant.

- Hydrogen Bonding: The multiple hydroxyl groups of H_3_AsO_3_ can readily form hydrogen bonds with the abundant oxygen-containing functional groups (hydroxyl, carboxyl, carbonyl, ether linkages) on the lignin surface. This is a plausible primary interaction for a neutral species.
- Surface Complexation: Direct complexation of H_3_AsO_3_ with specific lignin sites, particularly phenolic hydroxyls, cannot be entirely ruled out, potentially involving ligand exchange.
- Oxidation to As(V) followed by Anionic Adsorption: While less commonly discussed for As(III) interaction with lignin compared to other reducing agents, there is a possibility of partial surface oxidation of As(III) to As(V) (which forms H_2_AsO_4_^−^/HAsO_4_^2−^ at pH 6). These arsenate anions could then adsorb via mechanisms similar to Cr(VI), i.e., electrostatic interaction or ligand exchange with protonated sites.

The low q_max_ of 1.16 mg/g for As(III) [18] indicates that interactions, while kinetically rapid (highest k_2_ value), are limited in extent, perhaps due to the specificity or number of sites suitable for binding a neutral molecule or its potential oxidized forms.

#### 3.3.4. Correlation Between Metal Properties and Adsorption Affinity

The distinct hierarchy in adsorption capacities observed for unmodified Sarkanda grass lignin towards the nine investigated pollutants—Cu(II) >> [Ni(II) ≈ Co(II) ≈ Cd(II) ≈ Pb(II) ≈ Zn(II) ≈ Fe(II)] >> [As(III) ≈ Cr(VI)]—is not arbitrary. It can be qualitatively, and in some aspects quantitatively, correlated with the fundamental physicochemical properties of the metal(loid) ions and their inherent tendencies to interact with the oxygen-rich functional groups (primarily carboxyl and phenolic hydroxyl) present in the lignin structure.

The most striking observation is the significantly higher adsorption capacities for all seven divalent cations (Cd(II), Co(II), Fe(II), Ni(II), Pb(II), Zn(II)) (Cd^2+^, Co^2+^, Cu^2+^, Fe^2^, Ni^2+^, Pb^2+^, Zn^2+^) compared to the metalloid As(III) (predominantly neutral H_3_AsO_3_ at pH 6) and the anion Cr(VI) (predominantly HCrO_4_^−^ at pH 5). This strongly underscores the critical role of electrostatic attraction and ion exchange mechanisms facilitated by lignin’s deprotonated acidic functional groups (COO^−^, -ArO^−^) at the operational pH range (pH 5–6.5).

The positive charge of these ions directly facilitates their attraction to negatively charged sites on the lignin surface. Furthermore, their ability to displace protons (H^+^) from carboxyl and phenolic hydroxyl groups via ion exchange is a primary driving force for their robust uptake. The formation of strong coordinate bonds (chelation) with multiple oxygen donor atoms on lignin further stabilizes their binding.

As a neutral molecule at pH 6, As(III) cannot readily participate in ion exchange or strong electrostatic attraction. Its comparatively lower adsorption capacity (1.16 mg/g) suggests reliance on weaker or less abundant interaction mechanisms, such as hydrogen bonding with lignin’s oxygen-containing groups or potentially surface complexation via its own hydroxyl groups. While kinetically rapid (highest k_2_ value), these interactions appear to saturate quickly at a low overall capacity.

In the case of chromium, as an anion, (HCrO_4_^−^), Cr(VI) requires protonated (positively charged) sites on the lignin for electrostatic attraction. While such sites can form at acidic pH (e.g., pH 5), their abundance or accessibility on this unmodified lignin appears limited, resulting in the lowest observed adsorption capacity (0.861 mg/g). Ligand exchange with surface hydroxyls or potential redox reactions are also possible but seem to contribute minimally to overall capacity.

While all studied divalent cations showed significant adsorption, nuances in their capacities (Cu(II) being notably higher) can be attributed to a combination of their intrinsic properties:

- Ionic Radius and Charge Density: Smaller ionic radii and higher charge densities can lead to stronger electrostatic interactions and more stable complex formation. However, this is often counter-balanced by hydration effects [31].
- Hydrated Ionic Radius: Metal ions exist as hydrated species in aqueous solution. The energy required for desolvation (removal of the hydration shell) before adsorption can be significant and varies between ions. Ions with larger hydrated radii might face greater steric hindrance when accessing binding sites within the lignin’s porous structure [32].
- Electronegativity and Lewis Acidity: Higher electronegativity (Pauling scale) generally correlates with a greater tendency to accept electrons and form stronger covalent/coordinate bonds. Metal ions act as Lewis acids, and their strength influences their ability to interact with Lewis base sites on lignin (e.g., oxygen atoms in -OH, -COOH, C=O) [33].
- Copper(II) (q_max_ = 22.12 mg/g) exhibits a significantly higher adsorption capacity than the other M^2+^ ions (~13–14 mg/g). This is a common observation in biosorption and can be attributed to several factors:
  - Jahn–Teller Effects (Distortion): The d^9^ electronic configuration of Cu(II) leads to Jahn–Teller distortion, allowing it to form more stable and often tetragonally distorted complexes, which may fit particularly well with the spatial arrangement of lignin’s binding sites [34,35].
  - Irving–Williams Series: This empirical series often predicts the relative stability of complexes formed by divalent transition metal ions. It generally follows the order of Mn(II) < Fe(II) < Co(II) < Ni(II) < Cu(II) > Zn(II), meaning that copper (Cu) complexes are generally the most stable, while zinc (Zn) complexes are the least stable. The exceptionally high stability of Cu(II) complexes aligns with its superior adsorption capacity on lignin, where complexation is a key mechanism [36].
- “Hard–Soft” Acid–Base (HSAB) Principle: Lignin’s oxygen-containing functional groups (-OH, -COOH) are considered “hard” Lewis bases. According to the HSAB principle, hard bases prefer to bind with hard Lewis acids. Most of the divalent metal ions studied (e.g., Fe^2+^, Co^2+^, Ni^2+^, Cu^2+^, Zn^2+^, and Cd^2+^ to some extent) have borderline to hard acid characteristics, making them suitable partners for lignin’s functional groups. Pb^2+^, being a softer acid, might exhibit slightly different binding preferences or strengths, though its capacity was comparable to other hard/borderline cations in this system [37,38].
- Hydrolysis Constants (pKa values for M(H_2_O)_x_^2+^): The tendency of metal ions to hydrolyze and form hydroxo-complexes can influence their speciation and adsorption. The operational pH was generally chosen to minimize precipitation, but subtle differences in hydrolysis behavior near the optimal pH could play a role [39].
- Coordination Number and Geometry Preferences: Different metal ions have preferences for specific coordination numbers (e.g., 4, 6) and geometries (e.g., tetrahedral, octahedral, square planar). The ability of lignin to provide a suitable coordination environment will influence binding affinity [40].

While capacities vary, the consistent adherence to pseudo-second-order kinetics for all nine pollutants suggests that the chemical interaction step is universally rate-limiting. The differences in k_2_ values, particularly the high rate for As(III) compared to the M^2+^ ions, are intriguing. For As(III), this might imply that the formation of hydrogen bonds or initial surface complexes, though leading to low overall capacity, occurs very rapidly with readily accessible sites. For the M^2+^ ions, the similar order of magnitude for k_2_ values (where available and excluding As(III)) suggests that the intrinsic chemical reaction rates for ion exchange or chelation, once the ion reaches the active site, are broadly comparable. Differences in diffusion rates (external film or intraparticle) prior to the chemical step could also influence the overall observed rate but are not directly reflected in the k_2_ of the Ho-McKay model, which focuses on the chemisorption step.

The consistent finding that pseudo-second-order Ho–McKay kinetics best describe the adsorption onto lignin for all nine pollutants aligns well with the majority of studies on heavy metal adsorption by various lignins and other biosorbents. This widespread applicability of the Ho–McKay model strongly suggests that chemisorption involving electron sharing or exchange is a common rate-limiting mechanism in lignin-metal interactions, irrespective of the specific lignin source or metal ion, provided suitable functional groups are present.

### 3.4. Direct Evidence of Adsorption from Surface Analysis

Beyond the insights derived from equilibrium and kinetic modeling, direct surface analysis of the Sarkanda grass lignin pre- and post-adsorption, coupled with innovative biological assessments, provides compelling corroborative evidence for the successful sequestration of heavy metal(loid) ions and offers further clues into the nature of the interaction.

Scanning Electron Microscopy (SEM) was consistently employed across studies involving Cr(VI), Cd(II), Cu(II), Co(II), Ni(II), and Fe(II) to visualize the physical state of the lignin adsorbent before and after contact with the metal ion solutions [19,22,23,24,26,27,28].

The SEM micrographs of the pristine Sarkanda grass lignin typically revealed the morphology of unmodified lignin, an agglomeration of somewhat irregularly shaped particles, often described as being in the micrometer range (e.g., ~4 µm agglomerates, with constituent particles potentially smaller) [19,23,26,28]. While the surfaces might appear somewhat dense in some images, the material’s known high porosity (74%) [19] implies a significant internal structure. After exposure to metal ion solutions and subsequent adsorption, noticeable changes in the surface morphology of the lignin particles were frequently observed. For instance, in the study with Cr(VI), the lignin surface appeared “less distinct” or “filled” [23].

For Cu(II), Co(II), Ni(II), and Fe(II), SEM images consistently showed that the “untreated Sarkanda grass lignin displays a concentration of (what appears to be) dirtied micrometer particles that are well separated in relation to the surface morphology of the lignin treated with [the metal ion]” or that the morphology “differs significantly” after contamination [24,25,26,27]. These visual alterations suggest that the metal species have not only adsorbed onto the external surfaces but have also potentially interacted with, or deposited within, the porous structure, leading to a modified surface texture or appearance. This visual confirmation supports the hypothesis of significant metal uptake and interaction with the lignin matrix.

Representative SEM micrographs (Figure 3) reveal particles that are somewhat irregular in shape and tend to form micrometer-sized agglomerates. While seemingly dense in some views, the reported high porosity suggests significant internal void space within these particles or agglomerates. Optical microscopy further corroborated the particulate nature [21]. This morphology, combining an external surface with internal porosity, dictates the interface available for interaction with metal ions in solution [26].

Energy Dispersive X-ray (EDX) analysis, often coupled with SEM, provided direct elemental evidence of metal adsorption. For Cd(II) [19], Ni(II) [27], and by implication from descriptions for other metals, distinct peaks corresponding to the adsorbed metal were clearly identified in the EDX spectra of the lignin samples after exposure to the metal solutions, whereas these peaks were absent in the spectra of the virgin lignin. This unequivocally confirms the transfer of the target metal ions from the aqueous phase onto the solid lignin adsorbent.

**Figure 4:**
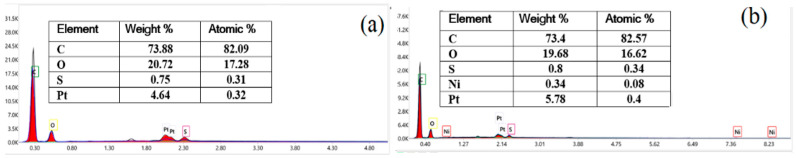
The EDX elemental analysis for lignin before adsorption (**a**) and after Ni(II) adsorption (**b**). Reproduced from [27], MDPI, 2024.

The EDX spectra generally showed prominent peaks for carbon (C) and oxygen (O), characteristic of lignin, alongside the peak for the adsorbed metal, as seen in Figure 4 [27]. In some cases, sulfur (S) was also detected, likely originating from the lignin structure itself or the metal salt (e.g., sulfates), and platinum (Pt) when samples were metallized for improved imaging [19,23,26,27].

In the comprehensive study on Cd(II) adsorption [19], contact angle measurements provided further insights into how the lignin surface properties were altered by metal uptake.

The unmodified Sarkanda grass lignin exhibited a hydrophilic character, with a contact angle below 90 degrees, indicating its tendency to absorb water. This is consistent with the presence of numerous polar functional groups (hydroxyl, carboxyl) on its surface.

Importantly, after adsorption of Cd(II) and reaching saturation, the Cd-loaded lignin displayed a hydrophobic character, with a contact angle exceeding 90 degrees. This shift from hydrophilic to hydrophobic strongly suggests that the active, polar binding sites on the lignin surface became occupied or masked by the adsorbed cadmium species. This not only provides indirect evidence for substantial surface coverage but also implies that the nature of the lignin surface was fundamentally altered by the chemisorption process, potentially influencing its interaction with the surrounding aqueous media environment.

### 3.5. Thermodynamic Insights

Thermodynamic studies were conducted for Cr(VI) [21,22], Cd(II) [19,20], and Cu(II) [23], providing insights into the spontaneity and energetic nature of their adsorption onto Sarkanda grass lignin (Table 7).

For all three metals, negative Gibbs Free Energy (ΔG) values were reported across the tested conditions, indicating that the adsorption processes were spontaneous and thermodynamically favorable. The magnitudes (e.g., −26 to −38 kJ/mol for Cr(VI) and Cu(II); −25 to −37 kJ/mol for Cd(II)) suggested significant interactions beyond simple physisorption, consistent with chemisorption or strong physisorption/ion exchange.

For all three metals (Cr, Cd, Cu), positive Enthalpy (ΔH) values were obtained, signifying that the adsorption processes were endothermic. This implies that adsorption is favored by an increase in temperature (within the stability limits of lignin), possibly due to increased molecular motion, enhanced diffusion, or the energy required for processes like desolvation of ions or activation of lignin binding sites. Endothermic adsorption often suggests that energy is required to overcome activation barriers, such as the dehydration of metal ions or the adsorbent surface, or for the formation of new chemical bonds.

Positive Entropy (ΔS) values were consistently reported, indicating an increase in randomness or disorder at the solid–liquid interface during adsorption. This is often attributed to the release of ordered water molecules from the hydration shells of the metal ions and/or from the adsorbent surface upon complex formation, leading to a net increase in the overall entropy of the system.

### 3.6. Biological Assessment of Adsorption Efficiency and Ecotoxicity

**Figure 5:**
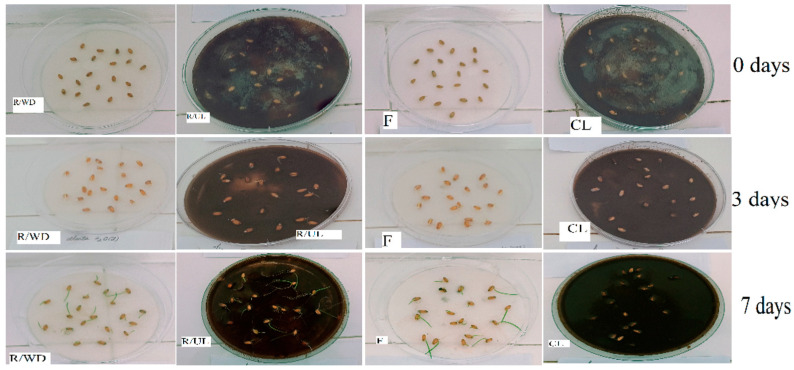
The germination of *Triticum aestivum* L. seeds over a period of 7 days, at an adsorption time of 60 min and a concentration of 58.693 mg/L Ni(II). Reproduced from [27], MDPI, 2024.

A unique and recurring feature across the investigations of Cr(VI), Cd(II), Cu(II), Co(II), Ni(II), and Fe(II) adsorption onto Sarkanda grass lignin was the use of phytotoxicity assays, specifically seed germination tests with *Triticum aestivum* L. (wheat, Glosa variety, seen in Figure 5) and *Lypercosium esculentum* (tomato, San Marzano variety) [19,22,23,24,26,27]. These biological tests served a dual purpose: to indirectly confirm the removal of metal ions from the solution and to assess the immobilization and sequestration of toxicity by the adsorbent.

Distilled water was used as a control for the filtrates, while uncontaminated lignin served as the control for the contaminated lignin samples. It is important to note that swollen, rotten, or moldy seeds at the end of the germination period were considered non-germinated. Figure 5 shows the germination of Glosa wheat seeds over a period of seven days for the following conditions: reference/uncontaminated lignin (R/UL), lignin contaminated with Ni(II) (CL), reference/distilled water (R/DW), and the filtrate (F) obtained after 60 min of adsorption at a Ni(II) concentration of 58.693 mg/L.

**Figure 6:**
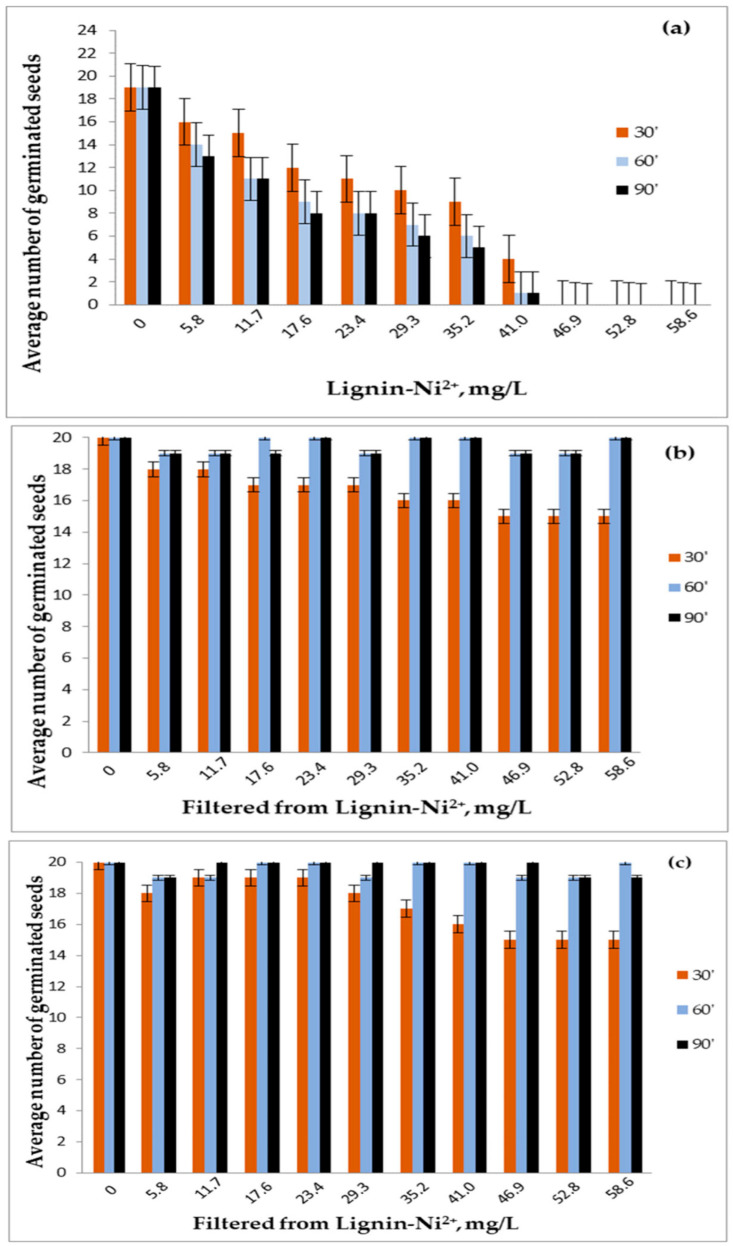
The average number of wheat seeds germinated at 3 days for the contaminated samples (**a**) and for the filtrates resulting from Ni(II) adsorption at 3 days (**b**) and 7 days (**c**) [27].

Figure 6a–c displays the average number of germinated wheat seeds after three repetitions at 3 days for samples contaminated with Ni(II). The average number of germinated seeds after three repetitions at 3 and 7 days for the filtrates resulting from the retention of Ni(II) at the three contact times between the phases is also shown.

From Figure 6a–c, the negative effect of metal ions on the germination of wheat caryopses can be observed, with this effect becoming more pronounced as both the metal ion concentration and the contact time between phases increase. This trend was also observed for the other studied metal ions.

In the control samples (uncontaminated lignin and distilled water), out of 20 seeds used, 19 germinated in the presence of uncontaminated lignin and 20 in distilled water. Notably, across all analyzed cases—for both wheat and tomato seeds—the average germination rate was 96.65% with uncontaminated lignin and 100% with distilled water.

For the filtrates, the number of germinated seeds after 3 days and the number of seedlings after 7 days was comparable to the control samples (distilled water and uncontaminated lignin) at contact times of 60 and 90 min. However, at the 30 min contact time, germination was reduced, suggesting that adsorption equilibrium had not yet been reached, and a longer contact time is required. This observation aligns with the conclusions drawn from the adsorption isotherms and kinetic parameters, which recommend an optimal contact time of 60 min.

Seven days after germination, in all samples treated with contaminated lignin—regardless of contact time or pollutant concentration—no additional seeds germinated, and the existing seedlings died. This confirms the strong adsorption capacity of lignin for the tested pollutant, as also demonstrated by the kinetic and equilibrium data.

Consistently, wheat and tomato seeds germinated significantly better (often comparable to the distilled water and uncontaminated lignin control samples) in the filtrates collected after the adsorption process, particularly at optimal contact times like 60 min. This demonstrated that the Sarkanda grass lignin effectively removed a substantial portion of the toxic metal ions from the aqueous phase, rendering the treated water less inhibitory to seed germination and early seedling development. For example, for most metals, germination energy and faculty in filtrates (especially from 60 and 90 min contact times) were close to control values, while those from shorter contact times (e.g., 30 min) often showed reduced germination, correlating with incomplete adsorption at non-equilibrium conditions.

Conversely, when wheat seeds were exposed directly to the lignin samples after they had adsorbed the metal ions (the “contaminated lignin”), a significant inhibition of germination and seedling survival was observed, especially at higher initial metal concentrations. In many instances, no seeds germinated, or existing seedlings died on the metal-laden lignin, even when the corresponding filtrate supported good growth.

This strongly indicates that the metal ions were firmly bound or sequestered by the lignin matrix, retaining their inherent toxicity but being effectively removed from the solution phase. This sequestration supports the idea of chemical binding (chemosorption) rather than weak, reversible physical adsorption.

The biological assay results generally corroborate the findings from the chemical adsorption studies. For instance, the observation that optimal metal removal (and thus filtrate non-toxicity) was achieved around 60 min of contact time aligned well with the kinetic and equilibrium data identifying 60 min as the optimal adsorption period

Beyond the conventional physicochemical evaluation of adsorption performance through equilibrium and kinetic studies, a distinctive and valuable component of the research synthesized in this review involves the use of biological assays. Specifically, seed germination tests employing *Triticum aestivum* L. (wheat, Glosa variety seen in Figure 5) and *Lypercosium esculentum* (tomato, San Marzano variety) were consistently utilized across investigations involving Cd(II), Co(II), Cr(VI), Cu(II), Fe(II), and Ni(II) [13,16,17,18,20,21]. These bioassays serve as an integrated measure of the overall efficiency of the adsorption process, providing insights into both the reduction in aqueous phase toxicity and the stability of metal sequestration by the Sarkanda grass lignin.

#### 3.6.1. Evidence for Effective Pollutant Removal from Aqueous Phase (Filtrate Bioassays)

A consistent and compelling finding across all studies employing these bioassays was the significantly improved germination and seedling development in the filtrates obtained after adsorption, especially when compared to seeds exposed to initial (pre-adsorption) metal solutions of similar concentrations (though direct comparison to initial solutions was not always the explicit control in the provided summaries).

The biological performance in filtrates often mirrored the adsorption efficiency observed chemically. For instance, filtrates obtained after the optimal contact time of 60 min (or longer, e.g., 90 min) generally supported seed germination and seedling growth rates comparable to, or very close to, those observed in the distilled water control.

This indicates that the Sarkanda grass lignin effectively removed a substantial fraction of the dissolved toxic metal ions, thereby reducing the phytotoxicity of the aqueous phase.

Conversely, filtrates from shorter contact times (e.g., 30 min), where adsorption equilibrium had not yet been fully established, often showed reduced germination energy and faculty compared to the 60/90 min filtrates and the control. This aligns with the expectation that less metal had been removed from solution at these shorter durations, leaving the filtrate more toxic [3,5,6,15,17,19].

These observations provide strong biological validation that the adsorption process, as characterized by equilibrium and kinetic studies, translates directly into a measurable reduction in ecotoxicity in the treated solutions.

#### 3.6.2. Evidence for Metal Sequestration and Immobilization (Contaminated Lignin Bioassays)

In the case of contaminated lignin bioassays, the response of wheat seeds when directly exposed to the metal-loaded lignin adsorbent provided critical insights into the strength and stability of metal binding.

In stark contrast to the non-toxic filtrates, the lignin samples contaminated with Cd(II), Co(II), Cr(VI), Cu(II), Fe(II), or Ni(II) consistently exhibited a pronounced inhibitory effect on seed germination and seedling survival. This effect was generally dose-dependent, with higher initial metal concentrations (leading to higher loading on the lignin) resulting in more severe toxicity.

The fact that the metals, once adsorbed onto the lignin, still exerted a toxic effect when seeds were in direct contact demonstrates that the metals were indeed present on/in the adsorbent. More importantly, the significant difference in toxicity between the metal-loaded lignin and the corresponding (largely non-toxic) filtrate suggests that the metals were firmly sequestered by the lignin. If the binding were weak or easily reversible under the conditions of the germination test, one might expect some leaching of metals back into the moist environment of the Petri dish, leading to toxicity similar to that of a dilute metal solution. The observed pattern points towards a relatively stable immobilization of the metal ions, consistent with the chemisorption mechanisms proposed in the previous chapter.

It was frequently reported that even if some seeds managed to germinate initially on the contaminated lignin (especially at lower metal loadings or shorter prior contact times), the resulting seedlings often died within the 7-day observation period. This further underscores the persistent toxicity of the metal-laden adsorbent and the efficacy of the lignin in concentrating and holding onto the harmful metal species.

The results from these biological assessments provide an ecologically relevant complement to the physicochemical data. The reduction in filtrate toxicity directly reflects the *q* values and percentage removal determined analytically. The persistence of toxicity in the metal-loaded lignin supports the concept of strong, likely chemosorptive binding, as weak physisorption might not retain the metals effectively enough to cause such pronounced direct contact toxicity while simultaneously producing a non-toxic filtrate. The consistent correlation between the 60 min contact time being optimal for both achieving high metal removal (chemical data) and producing non-toxic filtrates (biological data) strengthens the overall conclusions regarding the process efficiency.

#### 3.6.3. Ecological Implications of Bioassay Findings

The consistent results from the seed germination bioassays provide a powerful, ecologically relevant layer of validation that complements the physicochemical adsorption data. The findings offer a dual perspective on the environmental impact of using Sarkanda grass lignin as an adsorbent.

First, the high germination rates and healthy seedling growth observed in the filtrates serve as direct biological proof of effective aqueous phase detoxification. By successfully removing the dissolved, bioavailable metal ions, the lignin treatment demonstrably reduces the immediate phytotoxicity of the contaminated water. This is a critical outcome, as it suggests that water treated with this bioadsorbent would pose a significantly lower risk to downstream aquatic ecosystems or if used for agricultural irrigation.

Second, the severe inhibition of germination and seedling death observed on the metal-loaded lignin highlights the stable sequestration and concentration of toxicity. This finding has significant ecological implications. It confirms that the binding is strong enough to prevent significant leaching under moist conditions, supporting the chemisorption mechanism. However, it also clearly demonstrates that the adsorbent, once used, becomes a concentrated solid waste. This underscores a critical aspect of any adsorption technology’s lifecycle: the ‘cleaned’ water comes at the cost of creating a ‘toxic’ solid. This finding powerfully reinforces the argument that for a truly sustainable and circular application, the long-term management of the spent adsorbent—either through safe, stabilized disposal or, preferably, through effective regeneration and metal recovery—is an important concern that must be addressed in future research.

Research into the regeneration of Sarkanda grass lignin is currently ongoing, and the preliminary results are promising. The lignin was regenerated using 1N HCl and subjected to three desorption–readsorption cycles, demonstrating the potential for reuse without significant loss in adsorption efficiency.

The sorption rate for the regenerated lignin remained high for each metal ion, with slight decreases across regeneration cycles (78% for As(III) to 92% for Cu(II) after the first cycle; 72% for As(III) to 86% for Cu(II) after the second; and 67% for As(III) to 79% for Cu(II) after the third cycle).

These results not only support the continued investigation into regeneration processes but also recommend Sarkanda grass lignin as an effective and sustainable alternative for the removal of heavy metal ions from aqueous environments.

### 3.7. Integration with Physicochemical Adsorption Data

Across all studies, unmodified Sarkanda grass lignin demonstrated notable adsorptive capabilities under optimized laboratory conditions, typically employing a 5 g/L adsorbent dose, an initial solution pH between 5.0 and 6.5, and achieving equilibrium within approximately 60 min at ambient temperatures (20–24 °C). A clear and consistent trend in maximum adsorption capacities (q_max_) emerged:

Cu(II) (22.12 mg/g) > [Ni(II) (13.74 mg/g) ≈ Co(II) (13.67 mg/g) ≈ Cd(II) (~13.4 mg/g) ≈ Pb(II) (13.21 mg/g) ≈ Zn(II) (13.19 mg/g) ≈ Fe(II) (13.06 mg/g)] >> As(III) (1.16 mg/g) > Cr(VI) (0.861 mg/g)

This hierarchy underscores a strong intrinsic affinity for Cu(II) and a significant, remarkably similar capacity for a broad range of other divalent cations, while its ability to sequester the metalloid As(III) and the anion Cr(VI) is markedly lower.

A cornerstone finding of this collective work is the universal applicability of the pseudo-second-order Ho–McKay kinetic model to describe the adsorption process for all nine pollutants. This suggests that chemisorption, involving the formation of chemical bonds through electron sharing or exchange between the adsorbate and active sites on the lignin, is the rate-determining step.

This is an indication of specific interactions. While the Ho–McKay model rate constants (k_2_) varied, with As(III) exhibiting an exceptionally high rate despite its low capacity (suggesting rapid interaction with a limited number of specific sites), the values for most divalent cations were broadly comparable.

Equilibrium data were frequently best described by the Freundlich isotherm model, indicating adsorption onto a heterogeneous surface with a varied distribution of binding site energies—a characteristic expected for a complex, amorphous polymer like lignin. The favorability of adsorption was consistently supported by the Freundlich parameters. Thermodynamic studies (for Cr, Cd, Cu) revealed spontaneous (ΔG < 0) and endothermic (ΔH > 0) adsorption processes, with an increase in entropy (ΔS > 0), suggesting that higher temperatures favor adsorption and that the process leads to increased disorder at the solid–liquid interface, likely due to desolvation effects.

Mechanistically, the adsorption behavior is intrinsically linked to the chemical attributes of the Sarkanda grass lignin, particularly its substantial carboxyl (3.3 mmol/g) and phenolic hydroxyl (1.7 mmol/g) content, and its overall chelating capacity (67.14 meq/100 g). For divalent cations, the primary pathways are ion exchange (M^2+^ displacing H^+^ from -COOH and Ar-OH groups) and complexation/chelation with oxygen donor atoms. The superior uptake of Cu(II) can be rationalized by principles such as the Irving–Williams series and its strong tendency to form stable complexes. For anionic Cr(VI), adsorption likely involves electrostatic attraction to protonated lignin sites at acidic pH, potentially coupled with surface complexation or reduction to Cr(III) [41]. The neutral As(III) species (as H_3_AsO_3_ at pH 6) is proposed to interact via hydrogen bonding or weaker surface complexation.

Corroborative evidence from SEM–EDX analyses consistently confirmed metal uptake and post-adsorption morphological changes on the lignin surface. Furthermore, the innovative and consistent use of seed germination bioassays provided compelling, ecologically relevant validation. These tests demonstrated both the effective detoxification of the aqueous phase (non-toxic filtrates) and the strong sequestration of metals by the lignin matrix (evident from the toxicity of metal-loaded lignin), supporting the chemosorptive nature of the binding and the reduction in metal bioavailability.

### 3.8. Comparative Context: Performance of Sarkanda Grass Lignin in Relation to Other Lignin-Based Adsorbents

Sarkanda grass lignin exhibited a notably high affinity for Cu(II) with a q_max_ of 22.12 mg/g. This is a strong performance for an unmodified lignin. For instance, Reddad‘s group (2002) [42] reported Cu(II) uptake of 24.39 mg/g on pulp from sugar beet residues, while another study on peat [43] found a q_max_ of 21.13 mg/g for Cu(II), placing lignin’s performance favorably. However, other biomass resources, such as sunflower stalks [44] and eucalyptus black liquor lignin [45], have shown significantly enhanced Cu(II) capacities, exceeding 40 mg/g, highlighting a potential pathway for improving adsorption if desired.

The q_max_ values observed for Sarkanda grass lignin for divalent cations like Cd(II) (~13.4 mg/g), Co(II) (13.67 mg/g), Fe(II) (13.06 mg/g), Ni(II) (13.74 mg/g), Pb(II) (13.21 mg/g), and Zn(II) (13.19 mg/g) are within a competitive range for unmodified lignins. For example, Demirbas [46] reported a q_max_ of 8.2–9.0 mg/g for Pb(II) and 6.7–7.5 mg/g for Cd(II) on lignin from beech and poplar wood modified by alkaline glycerol delignification, while Gloaguen (1997) [47] found a capacity of 12–15 mg/g for Cd(II) on bark from different conifer species, both comparable to Sarkanda lignin. Another study by Wu et al. (2018) [48] on lignin and lignin-derived biochars from reed showed the highest sorption capacity for Zn(II) for the pristine lignin (6.043 mg/g), 5–20 times higher than that of the biochars (0.291–0.815 mg/g). This suggests lignin inherently possesses a good density of accessible functional groups for these common divalent cations.

Sarkanda grass lignin’s q_max_ for Cr(VI) (0.861 mg/g) is relatively low. This is not uncommon for unmodified lignins, as their surfaces are often negatively charged at optimal Cr(VI) adsorption pH (acidic), thus offering limited sites for anionic chromate species (9). For instance, unmodified hardwood lignin extracted from Kraft lignin was reported to adsorb between 2.5 and 3 mg/g of Cr(VI) [49]. Effective Cr(VI) removal by lignin often requires chemical modification to introduce positively charged groups (e.g., sulfonate) in order to enhance biosorption capabilities, with studies [50] reporting capacities exceeding 30 mg/g for such modified lignins.

Similarly, the As(III) capacity of Sarkanda grass lignin (1.16 mg/g) is modest. As(III) primarily exists as a neutral species (H_3_AsO_3_) at the pH (around 6) often employed, making strong electrostatic interactions difficult. Few studies report high As(III) uptake by unmodified lignins; for example, lignocellulosic waste from eucalyptus bark fibers showed As(III) uptake around 0.944 mg/g [45]. Enhanced arsenic removal typically involves lignins functionalized by lignin biochar decorated with Zn derived from the impregnation reaction of ZnCl_2_ colloid and the pyrolysis process [51], which provide specific binding sites for arsenite/arsenate reporting a q_max_ of 1.5 mg/g and 18.3 mg/g, respectively.

The consistent finding that pseudo-second-order Ho–McKay kinetics best describe the adsorption onto Sarkanda grass lignin for all nine pollutants aligns well with the majority of studies on heavy metal adsorption by various lignins and other biosorbents [52]. This widespread applicability of the Ho–McKay model strongly suggests that chemisorption involving electron sharing or exchange is a common rate-limiting mechanism in lignin-metal interactions, irrespective of the specific lignin source or metal ion, provided suitable functional groups are present.

The observation that the Freundlich isotherm often provides a better or comparable fit to the adsorption data of various metal ions (e.g., Cd(II), Co(II), Ni(II), Fe(II), and As(III)) on lignin-based biosorbents is well documented in the literature. This trend reflects the inherent energetic heterogeneity of binding sites on complex natural polymers like lignin, where functional groups exist in diverse chemical environments, leading to a range of binding energies. Such heterogeneity contrasts with the Langmuir assumption of a completely homogeneous surface with identical binding sites [53].

Studies on diverse lignins, from technical Kraft lignins to lignins from agricultural residues, frequently report superior or good fits for the Freundlich model when adsorbing heavy metals, supporting the concept of multilayer adsorption on energetically non-equivalent sites. For example, it was found [21] that Ni(II) adsorption on Sarkanda grass lignin followed the Freundlich model, suggesting surface heterogeneity. Similarly, Dang et al. (2021) [54] reported that the Freundlich isotherm better described Zn(II) adsorption onto functionalized lignocellulose derived from biomass waste, indicating a multilayer adsorption, compared with the Langmuir model, which is based on a monolayer adsorption assumption.

However, for other systems, the Langmuir model has also provided reasonable to good fits, suggesting that under specific conditions or for particular metal–ligand interactions, monolayer adsorption on more uniform patches of the lignin surface may also play a significant role. For instance, Wu et al. (2008) [55] demonstrated that Cd(II), Cr(III), Cu(II), Pb(II), and Zn(II) adsorption on lignin isolated from black liquor fit the Langmuir model well, suggesting monolayer adsorption. Likewise, Vescovi et al. (2015) [56] observed that Cr(VI) adsorption on industrial lignins followed the Langmuir model, implying the predominance of uniform active sites.

These findings underscore the importance of considering both isotherm models in evaluating the adsorption behavior of metal ions on lignin-based biosorbents, as the suitability of each model can vary depending on the specific metal–ligand interactions and the physicochemical properties of the lignin.

The assertion that carboxyl (COOH) and phenolic hydroxyl (Ar–OH) groups in sulfonated kraft lignin play a pivotal role in cation binding is well supported by existing research in lignin chemistry [57]. These functional groups are critical for the cation exchange capacity and complexation sites in lignin-based biosorbents. Numerous studies have highlighted the significance of these acidic groups in enhancing the adsorption capacities of various lignins for metals such as Pb(II), Cu(II), and Cd(II). For instance, Guo et al. (2008) [58] demonstrated that lignin can adsorb Pb(II), Cu(II), Cd(II), Zn(II), and Ni(II) ions due to the presence of carboxylic and phenolic-type surface groups. Similarly, Lindholm-Lehto et al. (2019) [59] emphasized the role of phenolic and carboxyl groups in the adsorption of heavy metals onto lignin-based materials and their increased content increase the sorption potential for divalent metal ions, in special [60].

The maximum adsorption capacity of metals greatly depended on the pH of the solution [61]. The observed pH dependence of metal adsorption generally favoring weakly acidic to near-neutral conditions (pH 5–6.5) for divalent cations and more acidic conditions (pH ~5) for anionic Cr(VI) aligns well with established principles governing the surface chemistry of lignocellulosic materials and the speciation of metal ions in aqueous solution [62].

For cationic metal adsorption, the increasing uptake with rising pH (up to the point where metal hydroxide precipitation becomes significant) is primarily attributed to the deprotonation of acidic functional groups inherent in lignin, principally carboxyl (-COOH) and phenolic hydroxyl (Ar-OH) groups [63].

Lignin, being a complex polyphenolic macromolecule, contains these groups with varying pKa values [64]. The pH-dependent behavior observed for Sarkanda grass lignin is not unique but is governed by a fundamental principle of surface chemistry common to all lignocellulosic materials. As extensively reported in the literature [62], lignin’s polyphenolic structure contains numerous acidic groups with a range of pKa values. The general deprotonation equilibria for these key groups are

-COOH → -COO^−^ + H^+^

Ar-OH → Ar-O^−^ + H^+^

This progressive dissociation with increasing pH is what creates the negative surface potential responsible for cation binding [65]. At lower pH values, these functional groups remain largely protonated, and the increased concentration of H^+^ ions in solution effectively competes with metal cations for these binding sites, thus reducing adsorption efficiency.

Conversely, for anionic species like chromate (HCrO_4_^−^ at pH 5), adsorption is typically favored at lower pH values when specific ligand exchange mechanisms with surface hydroxyls or even redox interactions may contribute to Cr(VI) binding under these conditions [66]. 

This comparative discussion underscores that unmodified Sarkanda grass lignin performs competitively as a biosorbent, particularly for a range of divalent heavy metal cations, when compared to other bio-based materials reported in the literature. Its inherent functional group composition provides a solid foundation for these interactions. While its capacity for certain species like Cr(VI) and As(III) is limited in its native form, this is a common trait for unmodified lignins and points towards the necessity of targeted modifications if high efficiency for these specific pollutants is required. The consistent pseudo-second-order kinetics and frequent better fit of the Freundlich isotherm observed for Sarkanda grass lignin mirror broad trends in the field of biosorption by lignocellulosic materials.

It is important to contextualize these performance metrics within a scientifically appropriate framework. The comparative analysis in our research has deliberately focused on other unmodified, bio-based adsorbents, particularly raw lignins and agricultural wastes. This “apples-to-apples” comparison is essential for establishing the intrinsic, baseline adsorption capabilities of native Sarkanda grass lignin. Our findings show that it is a highly competitive material within this class.

A direct comparison to highly engineered, commercially established adsorbents, such as activated carbons or optimized synthetic ion-exchange resins—which often exhibit capacities an order of magnitude higher—would be methodologically inappropriate at this stage. These materials are the product of energy-intensive activation or chemical functionalization processes that have been optimized over decades. The true scientific progression for a novel biomaterial involves: (1) first, establishing a robust baseline performance, as this study has done; (2) second, exploring targeted modifications to enhance that performance, as proposed in our Future Perspectives; and (3) finally, benchmarking the optimized and modified material against these commercial standards.

Therefore, this work provides the critical performance baseline necessary to guide and accurately quantify the improvements achieved through future research. It demonstrates that unmodified Sarkanda grass lignin is a high-performing starting material, not an end-product, with significant potential for further development into a competitive, sustainable adsorbent.

## 4. Conclusions and Future Perspectives

### 4.1. Overall Significance

The comprehensive body of research synthesized in this review systematically evaluates the potential of unmodified Sarkanda grass lignin as a biosorbent for a diverse suite of nine heavy metal(loid) ions: As(III), Cd(II), Cr(VI), Co(II), Cu(II), Fe(II), Ni(II), Pb(II) and Zn(II). This focused investigation, employing consistent methodologies, provides valuable insights into the adsorption behavior, mechanistic pathways, and potential applicability of this readily available biowaste material for environmental remediation.

Across all studies, unmodified Sarkanda grass lignin demonstrated notable adsorptive capabilities under optimized laboratory conditions, typically employing a 5 g/L adsorbent dose, an initial solution pH between 5.0 and 6.5, and achieving equilibrium within approximately 60 min at ambient temperatures (20–24 °C). A clear and consistent trend in maximum adsorption capacities (qmax) emerged from Cu(II) (22.12 mg/g) to Cr(VI) (0.861 mg/g)

This hierarchy highlights a strong inherent preference for Cu(II) and a notably consistent ability to bind various other divalent cations, whereas its capacity to capture the metalloid As(III) and the anion Cr(VI) is significantly lower.

The maximum adsorption capacity of metals greatly depended on the pH of the solution [62]. The observed pH dependence of metal adsorption generally favoring weakly acidic to near-neutral conditions (pH 5–6.5) for divalent cations and more acidic conditions (pH ~5) for anionic Cr(VI)—aligns well with established principles governing the surface chemistry of lignocellulosic materials and the speciation of metal ions in aqueous solution [63].

For cationic metal adsorption, the increasing uptake with rising pH (up to the point where metal hydroxide precipitation becomes significant) is primarily attributed to the deprotonation of acidic functional groups inherent in lignin, principally carboxyl (COOH) and phenolic hydroxyl (Ar-OH) groups [65]. At lower pH values, these functional groups remain largely protonated, and the increased concentration of H^+^ ions in solution effectively competes with metal cations for these binding sites, thus reducing adsorption efficiency. Conversely, for anionic species like chromate (HCrO_4_^−^ at pH 5), adsorption is typically favored at lower pH values when specific ligand exchange mechanisms with surface hydroxyls or even redox interactions may contribute to Cr(VI) binding under these conditions [60].

This comparative discussion underscores that unmodified Sarkanda grass lignin performs competitively as a biosorbent, particularly for a range of divalent heavy metal cations, when compared to other bio-based materials reported in the literature. Its inherent functional group composition provides a solid foundation for these interactions. While its capacity for certain species like Cr(VI) and As(III) is limited in its native form, this is a common trait for unmodified lignins and points towards the necessity of targeted modifications if high efficiency for these specific pollutants is required. The consistent Ho–McKay kinetics and frequent better fit of the Freundlich isotherm observed for Sarkanda grass lignin mirror broad trends in the field of biosorption by lignocellulosic materials.

The observation that the Freundlich isotherm often provides a better or comparable fit to the adsorption data of various metal ions (e.g., Cd(II), Co(II), Ni(II), Fe(II), and As(III)) on lignin-based biosorbents is well documented in the literature. This trend reflects the inherent energetic heterogeneity of binding sites on complex natural polymers like lignin, where functional groups exist in diverse chemical environments, leading to a range of binding energies. Such heterogeneity contrasts with the Langmuir assumption of a completely homogeneous surface with identical binding sites [61,64].

### 4.2. Concluding Remarks

This comprehensive series of studies establishes unmodified Sarkanda grass lignin as a promising, low-cost, and eco-friendly bioadsorbent with demonstrated efficacy, particularly for the removal of divalent heavy metal cations from aqueous solutions. The findings provide strong support for our guiding hypotheses, confirming the material’s predictable performance and mechanistic consistency.

Specifically, our first hypothesis—regarding the adsorbent’s performance and selectivity—was clearly verified. We established a distinct and consistent adsorption capacity hierarchy (Cu(II) > other M^2+^ >> As(III) > Cr(VI)), demonstrating that the lignin’s inherent physicochemical properties do indeed lead to a significant but highly selective uptake of diverse pollutants. The performance of this unmodified lignin for ions like Cu(II), Pb(II), Cd(II), and others is significant and positions it as a viable candidate material, especially considering its origin as an abundant agro-industrial byproduct, aligning well with the principles of a circular bioeconomy and waste valorization. While the capacities for certain challenging pollutants like Cr(VI) and As(III) are modest for this unmodified form, this finding itself is a critical part of the validated performance profile, providing a crucial baseline for future modification studies.

Furthermore, our second hypothesis—proposing a unified mechanistic pathway—was also robustly supported. The universal applicability of the pseudo-second-order kinetic model across all nine chemically diverse pollutants is a notable and powerful finding. This underscores the chemical nature of the dominant interactions and confirms that chemisorption acts as the common rate-limiting step, validating the concept of a unified reactive behavior of the lignin’s surface.

The successful demonstration of pollutant sequestration and reduction in aqueous toxicity through biological assays adds a unique and valuable dimension to its assessment, highlighting its potential to mitigate environmental risks.

Additionally, this work complements a growing body of research exploring the valorization of various biomass-derived components—such as black liquor lignins [65], hemicelluloses [66], and cellulose-rich residues [67]—as effective biosorbents. Studies have demonstrated that these biomaterials, either in raw or modified form, can offer enhanced adsorption capacities for a wide spectrum of pollutants, particularly heavy metals and dyes. Integrating unmodified Sarkanda grass lignin with other such bio-derived materials may offer synergistic benefits, opening new avenues for hybrid adsorbent development within sustainable water treatment frameworks.

Such integrative approaches align with the principles of waste valorization, resource recovery, and bio-based circular economy, presenting promising strategies for scalable and eco-efficient pollution mitigation.

Building upon the foundation laid by these investigations, several avenues for future research are pertinent to advance the practical application and deepen the understanding of Sarkanda grass lignin as an adsorbent:

1. Advanced Mechanistic Elucidation: While chemisorption is evident, employing advanced spectroscopic techniques (e.g., FTIR, XPS, NMR) pre- and post-adsorption for each metal could provide more definitive evidence of the specific functional groups involved in binding, their coordination environments, and the nature of the metal–lignin bonds. This could further explain the observed affinity differences (e.g., the Cu(II) preference).
2. Regeneration, Reusability, and Circularity: To fully realize the sustainability potential of Sarkanda grass lignin, comprehensive regeneration and reusability studies are important. The current work establishes its efficacy for a single use; future research must focus on developing effective and environmentally benign methods for desorbing the bound metal ions to regenerate the active sites. Potential strategies could involve washing with dilute acidic solutions (e.g., HCl, HNO_3_) to displace the metal cations via ion exchange with protons, or using chelating agents (e.g., EDTA) to strip the metals. The success of regeneration, the number of cycles the adsorbent can withstand without significant loss of capacity, and the management of the concentrated metal-rich eluate are all critical factors that will determine the material’s economic viability and its true place in a circular economy. The toxicity of the metal-loaded lignin, as demonstrated in our bioassays, underscores the importance of either safe disposal or, preferably, effective regeneration for sustainable application.
3. Performance in Complex Systems: Real-world wastewaters are complex matrices containing multiple competing ions, organic matter, and varying ionic strengths. Future studies should focus on:
  - Competitive Adsorption: Evaluating the selectivity of Sarkanda grass lignin in multi-metal systems.
  - Real Wastewater Testing: Assessing performance using actual industrial or contaminated water samples.
4. Optimization of Adsorption Conditions and Influence of Ionic Strength: Further exploration of the effect of temperature (to validate the endothermic nature across all metals and find optimal ranges) is warranted. Critically, investigating the impact of ionic strength by adding a background electrolyte (e.g., NaCl) would provide deeper mechanistic insight. A significant decrease in cation adsorption with increasing ionic strength would confirm the major role of non-specific electrostatic interactions (outer-sphere complexation), while processes less affected by ionic strength would point towards strong, specific inner-sphere complexation. This is essential for assessing the adsorbent’s performance in real, high-salinity industrial wastewaters.
5. Process Scale-Up and Engineering: Transitioning from batch studies to continuous-flow column experiments is essential for evaluating dynamic adsorption behavior, breakthrough characteristics, and for designing practical treatment systems.
6. Targeted Lignin Modification: Based on the baseline performance of this unmodified lignin, targeted chemical modifications could be strategically designed to improve capacity or selectivity for specific pollutants, particularly those for which the unmodified form shows lower affinity (like Cr(VI) and As(III)). Such modifications could include amination to introduce positive charges for anion binding, phosphorylation, or grafting of specific chelating ligands. Critically, any such modification study must be accompanied by a thorough characterization of the resulting material, including a clear summary of morphological changes to assess alterations in particle size, porosity, and surface texture, and how these physical changes correlate with the enhanced adsorption performance.
7. Life Cycle and Economic Assessment: A thorough life cycle assessment (LCA) and techno-economic analysis would be important to evaluate the overall environmental benefits and financial viability of using Sarkanda grass lignin for wastewater treatment in comparison to conventional methods and other biosorbents.

In conclusion, the collective research reviewed herein provides compelling pertinent evidence for the utility of unmodified Sarkanda grass lignin as an effective biosorbent for a range of heavy metal ions. It also contributes to the field of sustainable materials and environmental remediation, offering a foundation for further research aimed at optimizing its performance and translating its potential into practical, eco-friendly water treatment solutions.

## Figures and Tables

**Table 1:** WHO permissible limits, sources, effects, and references for heavy metals and metalloids in drinking water [29].

Metal/Metalloid	Limited Concentration * (mg L^−1^)	Common Sources	Effects
Arsenic	0.01	Pesticides, mining, natural groundwater contamination, treated wood, glass, and electronics production wastes	Skin lesions, cancers (skin/lung/bladder), cardiovascular disease, neurotoxicity
Cadmium	0.003	Batteries, pigments, electroplating, phosphate fertilizers, smoking	Kidney damage, osteoporosis, lung cancer, hypertension, bone demineralization
Chromium	0.05	Leather tanning, industrial effluents, stainless steel production, wood preservatives	Lung cancer, dermatitis, liver/kidney damage, neurotoxicity
Cobalt	NS **	Alloys, batteries (Li-ion), mining, orthopedic implants	Cardiomyopathy, thyroid dysfunction, dermatitis, respiratory irritation
Copper	2.0	Plumbing pipes, mining, agricultural fungicides	Gastrointestinal distress, liver/kidney damage
Iron	2.0	Corroded pipes, natural deposits, industrial waste	Liver damage, diabetes, oxidative stress (hemochromatosis)
Nickel	0.07	Batteries, electroplating, stainless steel, volcanic emissions	Dermatitis, respiratory carcinogenicity, lung cancer
Lead	0.01	Leaded paints, old plumbing, recycling batteries, contaminated soil	Neurodevelopmental deficits (children), anemia, hypertension, renal failure
Zinc	3.0	Galvanized pipes, mining runoff, fertilizers	Gastrointestinal distress, vomiting, immune dysfunction, deficiency growth

* World Health Organization (WHO) recommended permissible limits for heavy metal ions in aqueous solutions. ** No specific WHO guideline for cobalt in drinking water; limits often based on national standards (e.g., 0.05 mg/L in EU).

**Table 2:** Aqueous solution concentrations (mg/mL) of the studied pollutant species [18,19,20,21,22,23,24,25,26,27,28].

Metal/ Metalloid	Concentration (mg/L)									
Arsenic	7.49	14.98	22.48	29.97	34.46	44.95	52.44	59.94	67.43	74.92
Cadmium	11.24	22.48	33.72	44.96	56.21	67.45	78.69	89.93	101.17	112.41
Chromium	5.20	10.40	15.60	20.08	25.00	31.50	36.40	41.60	46.80	52.00
Cobalt	5.89	11.39	17.68	23.60	29.50	35.40	41.30	47.19	53.09	58.99
Copper	6.36	12.71	19.07	25.42	32.67	38.13	44.49	50.84	57.20	63.55
Iron	5.58	11.17	16.75	22.34	27.92	33.50	39.09	44.67	50.25	55.84
Nickel	5.87	11.74	17.61	23.48	29.35	35.22	41.09	46.95	52.82	58.69
Lead	20.72	41.44	62.16	82.88	103.60	124.32	145.04	165.76	186.48	207.20
Zinc	6.54	13.08	19.61	26.15	32.69	39.23	45.77	52.30	58.84	65.38

**Table 3:** Pollutant solution pH and interfacial contact times [18,19,20,21,22,23,24,25,26,27,28].

Ionic Specie	Symbol	pH	Contact Time (min)				Reference
			30	60	90	120	
Arsenic	As(III)	6	30	60	-	120	[18]
Cadmium	Cd(II)	6.2	30	60	-	120	[19,20]
Chromium	Cr(VI)	5	30	60	-	120	[21,22]
Cobalt	Co(II)	5	30	60	90		[23]
Copper	Cu(II)	5	30	60	-	120	[24,25]
Iron	Fe(II)	6.5	30	60	90		[26]
Nickel	Ni(II)	5	30	60	90		[27]
Lead	Pb(II)	6	30	60	90		[28]
Zinc	Zn(II)	6	30	60	90		[28]

**Table 4:** Specific analytical techniques used for the quantification of the studied metal ions.

Ionic Specie	Symbol	Method	Indicator	λ, nm	Reference
Arsenic	As(III)	Inductively Coupled Plasma Optical Emission Spectrometry with Hydride Generation (HG-ICP-OES)	-	189.042	[18]
Cadmium	Cd(II)	Spectrophotometry	Xylenol orange	575	[19,20]
Chromium	Cr(VI)	Spectrophotometry	Diphenylcarbazide	545	[21,22]
Cobalt	Co(II)	Spectrophotometry	Rubeanic acid	580	[23]
Copper	Cu(II)	Spectrophotometry	Rubeanic acid	390	[24,25]
Iron	Fe(II)	Inductively Coupled Plasma Optical Emission Spectrometry (ICP-OES)	-	238.204	[26]
Nickel	Ni(II)	Spectrophotometry	Rubeanic acid	590	[27]
Lead	Pb(II)	Spectrophotometry	4-(2-pyridylazo)-resorcinol	530	[28]
Zinc	Zn(II)	Spectrophotometry	Xylenol orange	570	[28]

**Table 5:** Langmuir maximum adsorption capacities (‘q_max_’) and adsorption capacities (‘q_e_’) of unmodified Sarkanda grass lignin for various metal(loid) ions (at optimal pH and 60 min contact time).

Metal/Metalloid	Maximum Adsorption Capacity q_max_, mg·g^−1^	Adsorption Capacity q_e_, mg·g^−1^	Reference
Arsenic	1.16	1.07 (±0.08)	[18]
Cadmium	~13.4 *	13.43 (±0.21)	[19,20]
Chromium	0.841	0.84 (±0.16)	[21,22]
Cobalt	13.67	11.56 (±0.07)	[23]
Copper	22.12	11.44 (±0.11)	[24,25]
Iron	13.06	12.38 (±0.19)	[26]
Nickel	13.74	12.56 (±0.23)	[27]
Lead	13.21	12.07 (±0.09)	[28]
Zinc	13.19	11.91 (±0.14)	[28]

* Langmuir q_max_ for Cd(II) is estimated based on observed q and comparison with other divalent cations, as the primary Cd(II) paper emphasized Freundlich fit [19,20].

**Table 6:** Characteristic parameters of the Freundlich and Langmuir models for the adsorption of As(III), Cd(II), Cr(VI), Co(II), Cu(II), Fe(II), Ni(II), Pb(II), and Zn(II) from aqueous solutions onto Sarkanda grass lignin [18,19,20,21,22,23,24,25,26,27,28].

Pollutant	Times	Freundlich Model			Langmuir Model		
	(min)	R^2^	1/n	k_F_	R^2^	q_m_ (mg/g)	K_L_
As(III)	30	0.9930	0.3387	0.8645	0.8951	2.7070	0.2296
	60	0.9875	0.2323	1.0949	0.8251	2.8042	0.3145
	120	0.9888	0.2320	1.0960	0.8287	2.8145	0.3134
Cd(II)	30	0.9810	0.9125	0.8321	0.8863	2.4224	0.2457
	60	0.9726	0.9312	1.0432	0.8276	2.5562	0.3411
	120	0.9637	0.9041	1.0359	0.8219	2.7593	0.3528
Cr(VI)	30	0.9930	0.9387	0.8645	0.8951	2.7070	0.2296
	60	0.9875	0.9423	1.0949	0.8251	2.8042	0.3145
	120	0.9888	0.9112	1.0960	0.8287	2.8450	0.3134
Co(II)	30	0.9743	0.9014	2.1732	0.9061	13.2187	0.0703
	60	0.9826	0.9124	1.9342	0.8217	14.0061	0.0690
	90	0.9632	0.9281	1.9382	0.7324	14.1398	0.0651
Cu(II)	30	0.9969	0.9020	2.1421	0.9820	13.0204	0.0643
	60	0.9971	0.9394	2.0001	0.9854	13.1122	0.0638
	90	0.9982	0.9500	1.9643	0.9882	13.1872	0.0631
Fe(II)	30	0.9656	0.9142	2.0327	0.7852	12.8821	0.0693
	60	0.9789	0.9242	1.9856	0.8712	13.6117	0.0687
	90	0.9684	0.9411	1.9902	0.7931	13.7002	0.0641
Ni(II)	30	0.9822	0.9028	2.1586	0.9179	12.4073	0.0801
	60	0.9942	0.9205	2.0075	0.8920	12.5206	0.0795
	90	0.9844	0.9994	1.9776	0.7766	12.5398	0.0794
Pb(II)	30	0.9971	0.9028	2.1586	0.9822	13.0208	0.0651
	60	0.9987	0.9595	2.0070	0.9868	13.1233	0.0759
	90	0.9990	0.9694	1.9776	0.9899	13.2100	0.0754
Zn(II)	30	0.9982	0.5506	1.9286	0.9926	12.5470	0.0786
	60	0.9966	0.6061	1.9466	0.9917	13.1061	0.0754
	90	0.9952	0.6341	1.9460	0.9910	13.1926	0.0750

**Table 7:** Thermodynamic parameters calculated for the adsorption of Cr(VI), Cd(II), and Cu(II) ions from aqueous solutions onto Sarkanda grass lignin.

Pollutant	pH	Time (min)	ΔG (kJ/mol)	ΔH (kJ/mol)	ΔS (J/mol K)
Cr(VI)	2.09 5	30 60 90 30 60 90	−26.08 −27.94 −28.82 −30.76 −35.41 −38.14	12.96 11.37 11.82 15.34 14.25 15.01	98.17 88.72 93.14 111.02 132.24 124.38
Cd(II)	1.02 6.03	30 60 120 30 60 120	−25.29 −27.02 −27.98 −30.67 −36.28 −37.05	12.01 11.21 11.43 15.18 14.36 14.81	99.21 86.24 87.31 112.32 137.92 126.83
Cu(II)	2.11 5.02	30 60 90 30 60 90	−26.17 −28.04 −29.13 −31.31 −36.18 −37.99	13.07 11.98 12.92 15.02 13.95 14.93	97.76 88.06 92.89 110.28 131.47 12.86
